# Applications of 2D-Layered Palladium Diselenide and Its van der Waals Heterostructures in Electronics and Optoelectronics

**DOI:** 10.1007/s40820-021-00660-0

**Published:** 2021-06-14

**Authors:** Yanhao Wang, Jinbo Pang, Qilin Cheng, Lin Han, Yufen Li, Xue Meng, Bergoi Ibarlucea, Hongbin Zhao, Feng Yang, Haiyun Liu, Hong Liu, Weijia Zhou, Xiao Wang, Mark H. Rummeli, Yu Zhang, Gianaurelio Cuniberti

**Affiliations:** 1 Institute of Marine Science and Technology, Shandong University, Qingdao, 266237 People’s Republic of China; 2 College of Energy Soochow Institute for Energy and Materials Innovations, Soochow University, Suzhou, 215006 People’s Republic of China; 3 Key Laboratory of Advanced Carbon Materials and Wearable Energy Technologies of Jiangsu Province, Soochow University, Suzhou, 215006 People’s Republic of China; 4 Centre of Polymer and Carbon Materials, Polish Academy of Sciences, M. Curie Sklodowskiej 34, 41-819 Zabrze, Poland; 5 Institute for Complex Materials, IFW Dresden 20 Helmholtz Strasse, 01069 Dresden, Germany; 6 Institute of Environmental Technology VŠB-Technical University of Ostrava, 17. listopadu 15, Ostrava, 708 33 Czech Republic; 7 Collaborative Innovation Center of Technology and Equipment for Biological Diagnosis and Therapy in Universities of Shandong, Institute for Advanced Interdisciplinary Research (iAIR), University of Jinan, Shandong, Jinan 250022 People’s Republic of China; 8 State Key Laboratory of Crystal Materials, Center of Bio and Micro/Nano Functional Materials, Shandong University, 27 Shandanan Road, Jinan, 250100 People’s Republic of China; 9 Department of Chemistry, Guangdong Provincial Key Laboratory of Catalytic Chemistry, Southern University of Science and Technology, Shenzhen, Guangdong 518055 People’s Republic of China; 10 State Key Laboratory of Advanced Materials for Smart Sensing, GRINM Group Co. Ltd., Xinwai Street 2, Beijing, 100088 People’s Republic of China; 11 Shenzhen Institutes of Shenzhen Institutes of Advanced Technology, Chinese Academy of Sciences, 1068 Xueyuan Avenue, Shenzhen University Town, Shenzhen, 518055 People’s Republic of China; 12 Institute for Materials Science and Max Bergmann Center of Biomaterials, Technische Universität Dresden, 01069 Dresden, Germany; 13 Center for Advancing Electronics Dresden, Technische Universität Dresden, 01069 Dresden, Germany; 14 Dresden Center for Computational Materials Science, Technische Universität Dresden, 01062 Dresden, Germany; 15 Dresden Center for Intelligent Materials (GCL DCIM), Technische Universität Dresden, 01062 Dresden, Germany

**Keywords:** Palladium diselenide, nTMDC, Synthesis, Field-effect transistors, Photodetectors, Sensors

## Abstract

The rapid development of two-dimensional (2D) transition-metal dichalcogenides has been possible owing to their special structures and remarkable properties. In particular, palladium diselenide (PdSe_2_) with a novel pentagonal structure and unique physical characteristics have recently attracted extensive research interest. Consequently, tremendous research progress has been achieved regarding the physics, chemistry, and electronics of PdSe_2_. Accordingly, in this review, we recapitulate and summarize the most recent research on PdSe_2_, including its structure, properties, synthesis, and applications. First, a mechanical exfoliation method to obtain PdSe_2_ nanosheets is introduced, and large-area synthesis strategies are explained with respect to chemical vapor deposition and metal selenization. Next, the electronic and optoelectronic properties of PdSe_2_ and related heterostructures, such as field-effect transistors, photodetectors, sensors, and thermoelectric devices, are discussed. Subsequently, the integration of systems into infrared image sensors on the basis of PdSe_2_ van der Waals heterostructures is explored. Finally, future opportunities are highlighted to serve as a general guide for physicists, chemists, materials scientists, and engineers. Therefore, this comprehensive review may shed light on the research conducted by the 2D material community.

## Highlights

- The structure–property relationship of PdSe_2_ is discussed, i.e., layer number vs. tunable bandgap, pentagonal structure vs. anisotropy-based polarized light detection.
- The synthesis approaches of PdSe_2_ are thoroughly compared, including bottom-up methods such as chemical vapor transport for bulk crystals, chemical vapor deposition for thin films and single-crystal domains, selenization of Pd films. Besides, top-down strategies are discussed, covering the mechanical exfoliation of bulk crystals, plasma thinning, and vacuum annealing as well as phase transition.
- The emerging devices of PdSe_2_ and its van der Waals heterostructures have been delivered such as metal/semiconductor contact, Schottky junction transistors, field-effect transistors, photodetectors, *p*–*n* junction-based rectifiers, polarized light detector, and infrared image sensors.
- Future opportunities of PdSe_2_-based van der Waals heterostructures are given including logic gate-based digital circuits, RF-integrated circuits, Internet of Things, and theoretical calculation as well as big data for materials science.

## Graphical abstract

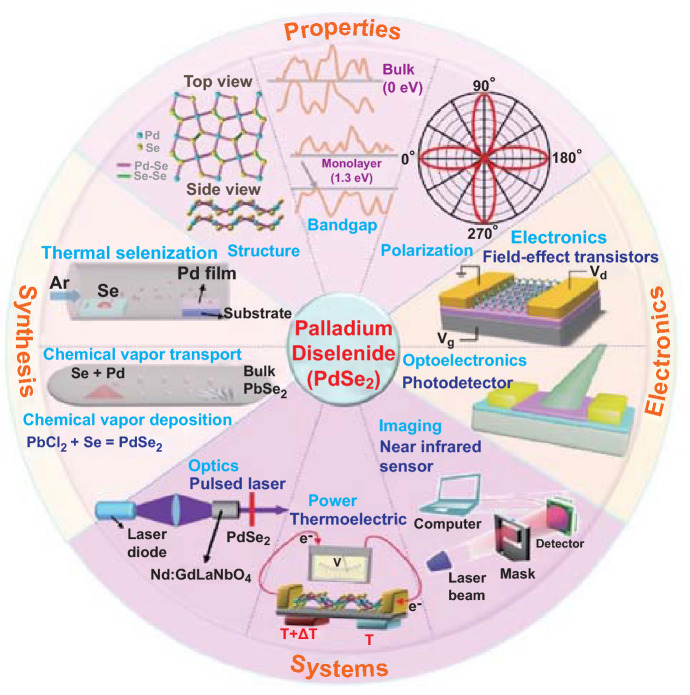

## Introduction

Significant research has been conducted on two-dimensional (2D) materials, including conductors (graphene) [1], semiconductors (MoS_2_), superconductors (NbSe_2_), and insulators (h-BN). The family of 2D-layered materials, possessing unique structures and extraordinary physical and chemical properties, has been continuously expanded with the addition of members such as transition-metal dichalcogenides (TMDCs) [2], phosphorene, borophene, and MXenes. These 2D materials have been widely employed in biomedical engineering [3], electronics and optoelectronics, photonics, optics, and related devices. Besides, 2D materials have boosted the field of smart sensing such as gas sensors [4]. They exhibit significant potential in devices such as photodetectors and photovoltaic cells; this is attributed to their distinct resonance absorption in the visible to near-infrared spectrum.

The family of TMDCs is an important component of 2D materials with a general formula of MX_2_, where M is a transition element and X is a chalcogen element. According to the International Union of Pure and Applied Chemistry (IUPAC) [5], transition elements generally comprise those from group 3 to group 12. TMDCs exhibit remarkable properties such as tunable bandgap, stability in air, and good charge transport, which is of great significance to the development of modern technology. Currently, more commonly discussed TMDCs are group-6 TMDCs [6], which primarily include MoS_2_, MoSe_2_, MoTe_2_, WS_2_, WSe_2_, and WTe_2_. Recently, 2D TMDCs and their heterojunction have attracted more and more research interest in the field of broadband photodetectors due to their excellent electronic and optoelectronic properties and show broadband photodetection from UV to IR [7]. In fact, TMDCs have retained significant research value for fundamental physics and device applications.

### Emerging Noble Transition-Metal Dichalcogenides

Dichalcogenides of group-10 transition metals MX_2_ (M = Pd, Pt, X = S, Se, Te) have recently received increased research attention owing to their novel properties. They are often referred to as noble transition-metal dichalcogenides (nTMDCs) because all the metal elements in group 10 are noble metals [8]. Here, nTMDCs [9] primarily refer to PtS_2_, PdS_2_, PtSe_2_, and PdSe_2_, and they show a significant intrinsic nature resulting from rich *d*-electron content. Besides, PtTe_2_-based photodetectors demonstrate an air stable and high performance in MIR photodetection up to 10.6 µm [10].

The fundamental properties of the selected nTMDCs are listed in Table 1. The nTMDCs are, however, yet to be fully understood; therefore, there is much scope for research in this area.

**Table 1:** The basic properties of the noble transition-metal dichalcogenides group

Material types	Phase	Bandgap	Lattice parameters	Lattice structure	Crystal system	Space group	References
PdSe_2_	Marcasite	0 eV (bulk) 1.33 eV (1L)	*a* = 5.74 Å; *b* = 5.92 Å; *c* = 7.69 Å	Pentagonal	Orthorhombic	Pbca [61]	[35]
PdSe_2_	Marcasite		*a* = 5.79 Å; *b* = 5.95 Å; *c* = 8.59 Å	Pentagonal	Orthorhombic	Pbca [61]	[132]
PdSe_2_	1 T	0 eV (≥ 2L) 0.778 eV (1L)	*a* = 3.73 Å; *c* = 4.79 Å	n.a	Hexagonal		[133]
PdSe_2_	2H	n.a	*a* = 3.58 Å; *c* = 10.90 Å	n.a	Hexagonal		[133]
PdSe_2_	Pyrite	n.a	a = 5.74 Å; *b* = 5.86 Å; *c* = 7.53 Å	n.a	Orthorhombic		[133]
PdSe_2_	Marcasite	n.a	*a* = 5.06 Å; *b* = 6.12 Å; *c* = 3.89 Å	n.a	Orthorhombic		[133]
PdS_2_	Marcasite		*a* = 5.50 Å; *b* = 5.59 Å; *c* = 8.61 Å	pentagonal	Orthorhombic	Pbca [61]	[134]
PdS_2_	1 T	0 eV (≥ 2L) 1.1 eV (1L)	*a* = 3.068 Å	n.a	Hexagonal	Pbca [61]	[14]
PdS_2_	2H	n.a	*a* = 3.82 Å; *c* = 9.33 Å	n.a	Hexagonal	n.a	[133]
PdS_2_	Pyrite	0 eV (Bulk) 1.399 eV (1L)	*a* = 5.45 Å; *b* = 5.53 Å; *c* = 7.20 Å	n.a	Orthorhombic	n.a	[133]
PdS_2_	Marcasite	n.a	*a* = 4.78 Å; *b* = 5.67 Å; *c* = 3.79 Å	n.a	Orthorhombic	n.a	[133]
PdTe_2_	Merenskyite	n.a	n.a	n.a	Trigonal	P-3m1 [164]	[135]
PdTe_2_	1 T	n.a	*a* = *b* = 4.0365 Å; *c* = 5.1262 Å	n.a	Hexagonal		[136]
PdTe_2_	2H	n.a	*a* = 3.83 Å; *c* = 11.60 Å	n.a	Hexagonal		[133]
PdTe_2_	Pyrite	0 eV (≥ 1L)	*a* = *b* = *c* = 6.54 Å	n.a	Orthorhombic		[133]
PdTe_2_	Marcasite	n.a	*a* = 5.40 Å; *b* = 6.65 Å; *c* = 4.10 Å	n.a	Orthorhombic		[133]
PtSe_2_	1 T	0 eV (bulk) 1.17 eV (1L)	*a* = *b* = 3.73 Å; *c* = 5.08 Å	Octahedral crystal	Hexagonal		[11]
PtS_2_	1 T	0.25 eV (bulk) 1.6 eV (1L)	*a* = *b* = 3.54 Å; *c* = 5.04 Å	Octahedral coordination structure	Hexagonal		[30]

Before introducing the PdSe_2_, we first look at the properties of other nTMDCs. PtS_2_ exhibits very strong interlayer interactions and layer-dependent indirect bandgaps ranging from 1.6 (monolayer) to 0.25 (bulk) eV. In recent years, few-layer PtS_2_ has become a promising material for field-effect transistors (FETs) with high mobility and on/off ratios. Furthermore, PtS_2_-based devices have demonstrated excellent performance with respect to photodetection and sensing. Similarly, 2D PtSe_2_ shows prominent layer-dependent properties, and the bandgap of monolayer PtSe_2_ is 1.2 eV, while that of bulk PtSe_2_ is zero. The carrier mobility of few-layer PtSe_2_ can theoretically exceed 10^3^ cm^2^ V^−1^ s^−1^, and very high stability in air is demonstrated [11]. Few-layered PtSe_2_ has been utilized in a variety of applications, such as FETs and photodetectors. PtSe_2_ shows good potential in piezoelectric devices, saturable absorbers, and electrochemical energy conversion. The structure of PdS_2_ comprises a pentagonal network, which includes two Pd atoms and three S atoms distributed on the atomic plane [12]. Monolayer PdS_2_ has two stable structures: one is a standard 1 T structure and the other involves a bulk-like geometry [13]. Through predictions and calculations, monolayer PdS_2_ has been determined to possess a semiconducting feature with a bandgap of approximately 1.1 eV, while bilayer PdS_2_ possesses a semimetallic feature [14]. Through first-principle calculations, a few-layer PdS_2_ has been predicted theoretically with good electronic and optoelectronic properties. However, few experimental synthesis studies have been reported in this regard. Thus far, there remain good opportunities for the material optimization and device applications of PdS_2_. But PdS_2_ pentagonal structure is not thermodynamically stable, which limits its applications. Hence, PdSe_2_ becomes of importance for exploiting the polarization properties and related optoelectronic applications.

### Importance of PdSe_2_

**Scheme 1:**
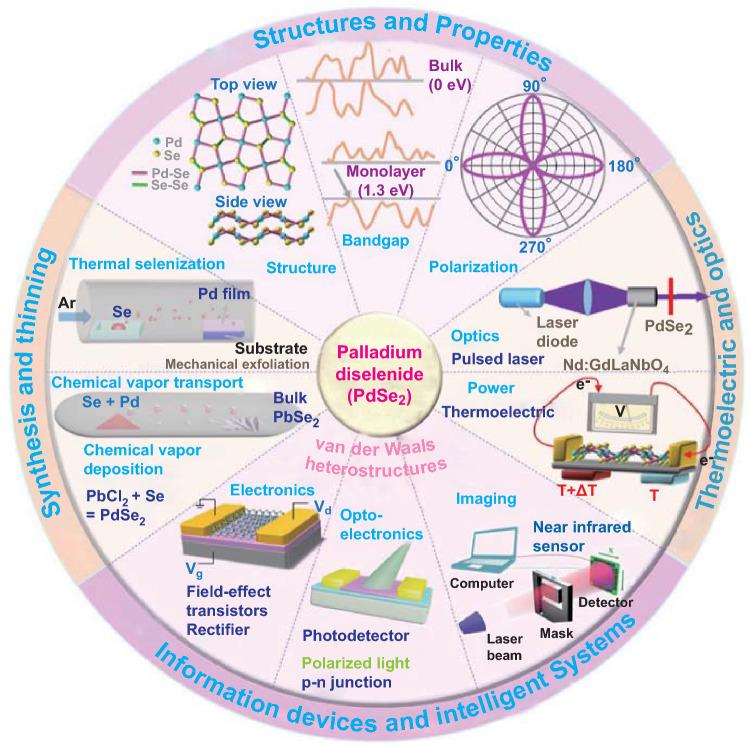
PdSe_2_ and its heterostructures for electronic, optic, and optoelectronic devices and systems

PdSe_2_ exhibits unique physical properties such as high carrier mobility, tunable bandgaps, and magnetic transport. PdSe_2_ has become a popular 2D material owing to its good stability [15], layer-dependent bandgap, and in-plane optical anisotropy [16]. PdSe_2_ (Scheme 1) has been integrated into electronic [17], thermoelectric, optical [18], and optoelectronic devices [19]. The diverse polymorphisms of PdSe_2_ provide the platform for investigating the topological states and the applications of quantum information devices [20].

PdSe_2_-based van der Waals heterostructures (vdWHs) have been widely incorporated in current rectifier, polarized light photodetector, and infrared image sensor applications. First, the direct synthesis of PdSe_2_-based vdWHs has been investigated via deposition of PdSe_2_ over other 2D materials such as graphene [21], MoS_2_ [22], MoSe_2_ [23], GeSe [24], and SnSe_2_. The stacking with arrayed nanomaterials gives rise to heterostructure devices such as ZnO nanorods and Si nanowires [25]. A perovskite [26] heterostructure can be formed with PdSe_2_ using a self-powered image sensor.

In this review, we discuss the most recent developments with regard to PdSe_2_ and its vdWHs, including approaches for its synthesis and its application in electronics, optoelectronics, and optics. We believe that this comprehensive contribution may attract the attention of research communities as well as industrial engineers interested in PdSe_2_ material development and device integration.

## Structure and Properties of PdSe_2_

This section introduces in detail the crystalline structure, electronic structure, energy band, vibrational phonon modes, and phase transition of PdSe_2_, which are the bases of its application in various fields.

### Crystal Structure

As a 2D-puckered pentagonal material, PdSe_2_ possesses orthorhombic lattices and a low symmetry, and it was identified as the first TMDC with a pentagonal structure [27]. The crystalline structure of PdSe_2_ has been studied from as early as 1952 [28], owing to which a good foundation for current research has been laid. Most recently, 2D materials with pentagonal structures have attracted much research attention. Examples include penta-graphene, penta-PdS_2_ [12], penta-SnS_2_, penta-silicene, and penta-germanene. The structures of these pentagonal materials differ from most hexagonal structures in 2D materials with high symmetry. They can still possess a relatively low symmetry in regular corrugated modes. Therefore, unique physical properties emerge with pentagonal structures, leading to novel electronic applications.

**Fig. 1:**
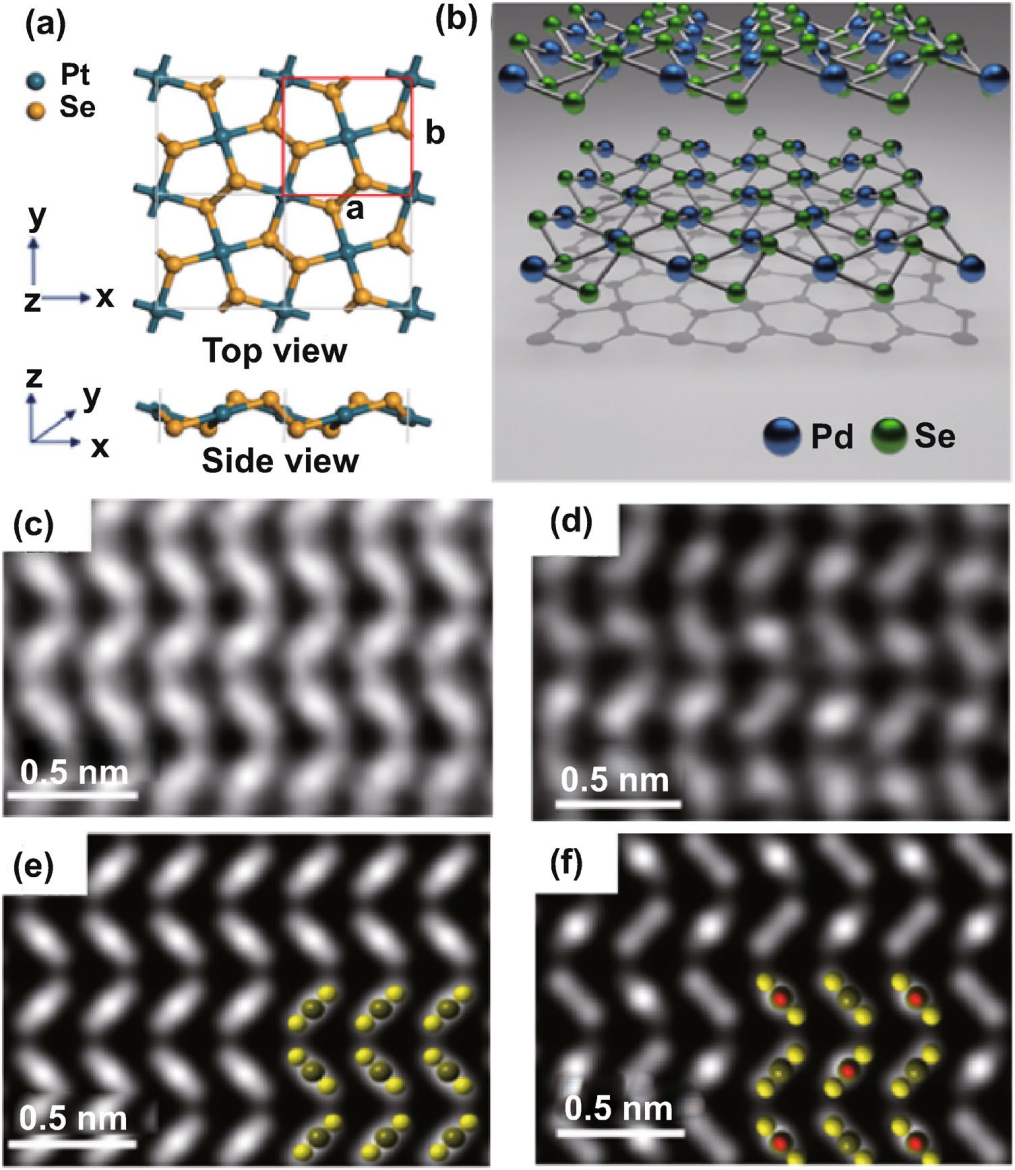
Atomic structure of PdSe_2_. **a** Top view and side view of penta-PdSe_2_ monolayers, where a unit cell is marked using a red line. The blue and yellow spheres represent the Pd and Se atoms, respectively. Reprinted with permission from Ref. [199]. Copyright 2015, Royal Society of Chemistry. **b** 3D crystallographic structure of puckered pentagonal PdSe_2_. **c**, **d** Z-contrast STEM images of PdSe_2_ crystal structure with even and odd numbers of layers. **e**, **f** Corresponding simulated images of PdSe_2_ crystals with even and odd numbers of layers. Insets in **e**, **f** display atomic models of the corresponding STEM images. Reprinted with permission from Ref .[15]. Copyright 2017, American Chemistry Society

Figure 1a shows the top and side views of the monolayer PdSe_2_ structure; it can be clearly seen that the one-unit cell contains four Pd atoms and eight Se atoms (top plane). In one PdSe_2_ layer, the two Se atoms cross the Pd layer in the form of a Se–Se dumbbell (bottom plane).

The uncommon layered structure is composed entirely of pentagonal rings, in which each Pd atom binds to four Se atoms, and two adjacent Se atoms form a covalent bond in one layer [23]. Hence, there exists no dangling bond in one PdSe_2_ layer, and these layers interact via van der Waals forces, resulting in excellent stability in air. The lattice parameters *a*, *b*, and *c* are, respectively, 5.75, 5.87, and 7.69 Å for PdSe_2_. Each layer of PdSe_2_ crystal has a vertical puckering height of 1.6 Å, where Pd atoms exhibit an unusual planar tetra-coordination [15].

Figure 1b shows the corresponding three-dimensional (3D) schematic of a monolayer PdSe_2_ structure from a projected top view and side view [15], which is similar to that of black phosphorus (BP).

Figure 1c, d exhibits the annular dark-field (ADF) image of the PdSe_2_ crystals, as generated via scanning transmission electron microscopy (STEM), as well as the corresponding image simulations (Fig. 1e, f) [15]. This approach can well prevent the formation of the disordered region of PdSe_2_ flakes due to the transfer process onto the TEM grid. As can be seen, owing to the difference in symmetry, the even and odd layers of PdSe_2_ flakes can give rise to a variation in the ADF images. Nonetheless, these patterns are in good agreement with the corresponding image simulations [15]. Moreover, the STEM images verify the puckered structure with waved Pd–Se layers of PdSe_2_.

The morphology and structure of PdSe_2_ have shown satisfactory property–structure correlation. Indeed, the anisotropic orientation of the PdSe_2_ domains results in polarized light detection [29]. The strain engineering influences the phonon response, which demonstrates its potential in the field of flexible electronics. Defect engineering such as vacancies could affect the air stability of the PdSe_2_ transistor as well as the Ohmic contact. The phase transition mechanism should be investigated for a better understanding, and more new phases of PdSe_2_ can be exploited for further applications. The high-pressure induced phase of PdSe_2_ renders a photovoltaic material. The hexagonal T phase of PdSe_2_ resulted in a high-efficiency solar cell. The pyrite phase PdSe_2_ exhibits superconductivity induced by high pressure.

Bulk PdSe_2_ crystals display D_2h_ point group symmetry and *Pbca* space group symmetry [28]. The pentagonal PdSe_2_ belongs to the phase of marcasite in the crystal system of orthorhombic [27]. By comparison, thin PdSe_2_ flakes with an odd number of layers are allocated to space group *P*2_1_/*c* (No. 14) and point group *C*_2*h*_ (2/*m*), which possess inversion symmetry, while thin PdSe_2_ flakes with an even number of layers are allocated to space group *Pca*2_1_ (No. 29) and point group C_2*v*_ (*mm*2), which do not possess inversion symmetry [15].

### Electron Orbital Properties

The conventional hexagonal structures are featured with isotropy, e.g., MoS_2_. The symmetrical hexagons lead to weak interlayer interaction due to the d^4^*sp* hybridization in TMDCs [20]. Here, the Mo and W elements are in lack of *d* orbital electrons. Besides, the *d* orbital of Pt atom and p_z_ orbital of S atom are hybridized into d^2^*sp*^3^ type, which accounts for the strong interlayer interaction in PtS_2_ [30].

But the hybridization between Pd and Se orbitals is complicated in PdSe_2_. First, one need to understand the electron configuration of these two elements. The Pd metal has a fully occupied *d* orbital with electron configuration of [Kr]4d^10^. And the Se is a *p*-block element, with an electron configuration of [Ar]3d^10^4s^2^4p^4^. In a single-layer PdSe_2_, one Pd atom is coordinated to four Se atoms, forming a square-planar structure [31]. Quite often, the Pd^2+^ results in the d^8^ configuration such as PdCl_2_. Therefore, the PdSe_2_ possesses a phase of marcasite analogous to the FeS_2_ [27]. The weak hybridization occurs between the 4d_z_^2^ orbitals of Pd atom and 4p_z_/3d_z_^2^ orbitals of Se atom, which led to the low symmetry [31].

The hybridization of Pd 4d orbit and Se 4p orbit has resulted in the covalent bond in PdSe_2_ [32]. The bands near Fermi level are contributed by the *p* orbitals of Se element. The conductance band minimum and valence band maximum of monolayer PdSe_2_ have stemmed from the *p* states of Se and *d* states of Pd. The spin–orbital coupling does not influence the electronic structure of monolayer PdSe_2_ [33]. But, with increasing the layer number, the interlayer coupling becomes strong and decreases the bandgap of bilayer and trilayer PdSe_2_ compared with monolayer PdSe_2_ [32]. Besides, the stacking types determine the bandgap of PdSe_2_, e.g., the AA and AB stacking for bilayer PdSe_2_ and the AAA, ABA, and ABB stacking for trilayer PdSe_2_ [32].

Indeed, the pentagonal PdSe_2_ is analogous to other puckered 2D materials, i.e., phosphorene and silicene, which feature with anisotropy [15]. The buckling of puckered 2D materials lead to a strong spin–orbital coupling between adjacent two layers, which is accounted for the topological quantum phase transition.

With the doping of transition-metal atoms such as Cr and Mn, new energy levels were introduced into the band structure of PdSe_2_ [34], which decrease its bandgap and introduce new spin nondegenerate states. These spin states around the Fermi level could cause the spin polarization.

After knowing the electron orbital theory, we now come to discuss the band structure of PdSe_2_.

### Electronic Band Structure

This section discusses the electronic energy band structures and density of states (DOSs) of PdSe_2_. Similar to that of most layered TMDCs, the indirect bandgap of PdSe_2_ largely depends on the number of layers.

**Fig. 2:**
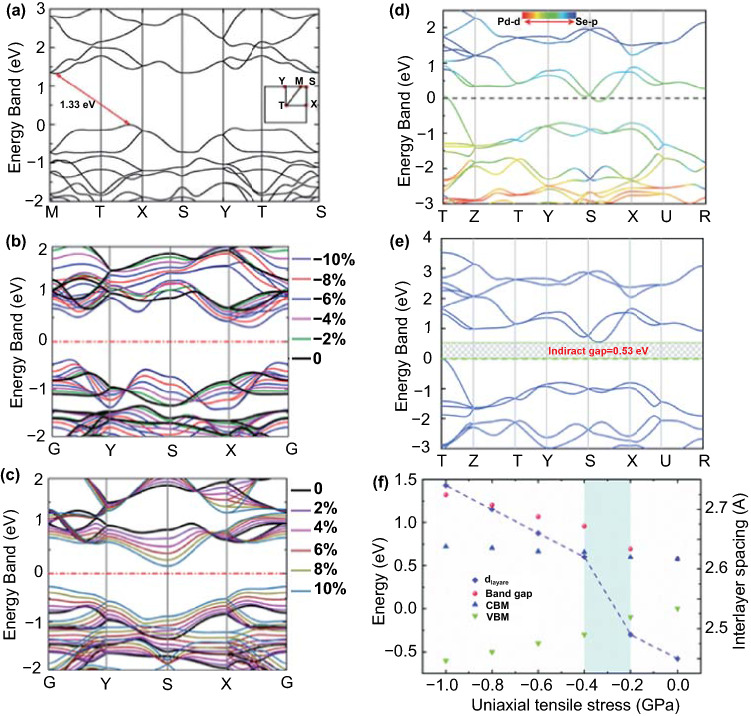
**a** Electronic band structure of monolayer PdSe_2_ with no strain. Reprinted with permission from Ref. [35]. Copyright 2018, Royal Society of Chemistry. Electronic band structure of monolayer PdSe_2_ with symmetrical biaxial **b** compressive, and **c** tensile strains. Reprinted with permission from Ref. [36]. Copyright 2018, American Chemistry Society. **d** Electronic band structure of bulk PdSe_2_, where the Fermi level is set to zero. The red and blue regions represent the contributions from Pd 4d and Se 4p states, respectively. **e** Electronic band structure of bulk PdSe_2_ under a tensile stress of 1.0 GPa. **f** Bandgap, CBM, VBM, and interlayer spacing (d_layers_) of bulk PdSe_2_ as a function of the uniaxial tensile stress, where the blue region presents the rapid increase of d_layers_. Reprinted with permission from Ref. [33]. Copyright 2019, Royal Society of Chemistry

The bandgap of PdSe_2_ has been calculated [33] via the approaches of generalized gradient approximation (GGA), density functional theory (DFT) of Perdew, Burke, and Ernzerhof (PBE). Here, the bandgap of PdSe_2_ is defined as the energy difference between the valence band (VB) and the conduction band (CB). The indirect bandgap of monolayer PdSe_2_ with semiconducting characteristics is 1.33 eV (Fig. 2a), and this decreases with the increase in the number of PdSe_2_ layers until the bulk PdSe_2_ has no bandgap (0 eV) with semimetallic characteristics (Fig. 2d).

In the cases of TMDCs and phosphorene, the valence band maximum (VBM) and conduction band minimum (CBM) are located along the high-symmetry lines. However, in the electronic structure of PdSe_2_ [35], VBM is located between the high-symmetry Γ and X, while the CBM is located between M and Γ (Fig. 2a).

Meanwhile, the effects of strain, particularly biaxial strains, have been investigated on the electronic and optical properties of PdSe_2_ [36]. Figure 2b, c shows the evolution of the monolayer PdSe_2_ energy bands under compressive and tensile strains, respectively. The black line represents the energy band of PdSe_2_ in the unstrained state, while the other colors represent the energy bands of PdSe_2_ in the various strained states. The compressive and tensile strains decrease the CBM and increase the VBM of monolayer PdSe_2_, and the VBM and CBM rise to a maximum value for compressive or tensile strains of –10%, leading to the minimum bandgap of monolayer PdSe_2_ [35]. Moreover, under compressive strain along the *x*-direction, the monolayer PdSe_2_ shows a negative Poisson’s ratio, possibly resulting from the Se–Se bond [37].

Figure 2d shows the energy band of bulk PdSe_2_, where the electronic structure shows a negative indirect bandgap with semimetallic characteristics at the DFT level. However, VB and CB are not entangled around the Fermi level [33]. A semimetallic feature of bulk PdSe_2_ can be observed through ultraviolet photoemission spectroscopy [26] and optical absorption [25]. However, bulk PdSe_2_ exhibits semiconducting characteristics from resistivity experiments [38]. Hence, further research is necessary to understand the bandgap of bulk PdSe_2_ better owing to this contradiction.

Figure 2e reveals the electronic band structure of bulk PdSe_2_ calculated via DFT under the tensile stress of –1.0 GPa, whereby a bandgap of 0.48 eV is observed. When uniaxial tensile stress is applied to bulk PdSe_2_ along the out-of-plane direction, the lattice parameter *c* and interlayer distance increase [33]. In orthorhombic PdSe_2_, the bandgap is positively correlated with the interlayer distance, indicating that the interlayer interaction has a significant influence on the electronic structure. Figure 2f shows the interlayer spacing (d_layers_) and bandgap of bulk PdSe_2_ as a function of the uniaxial tensile stress. As the interlayer spacing increases, VBM decreases dramatically, while CBM increases slightly, resulting in an increase in the bandgap of bulk PdSe_2_.

**Fig. 3:**
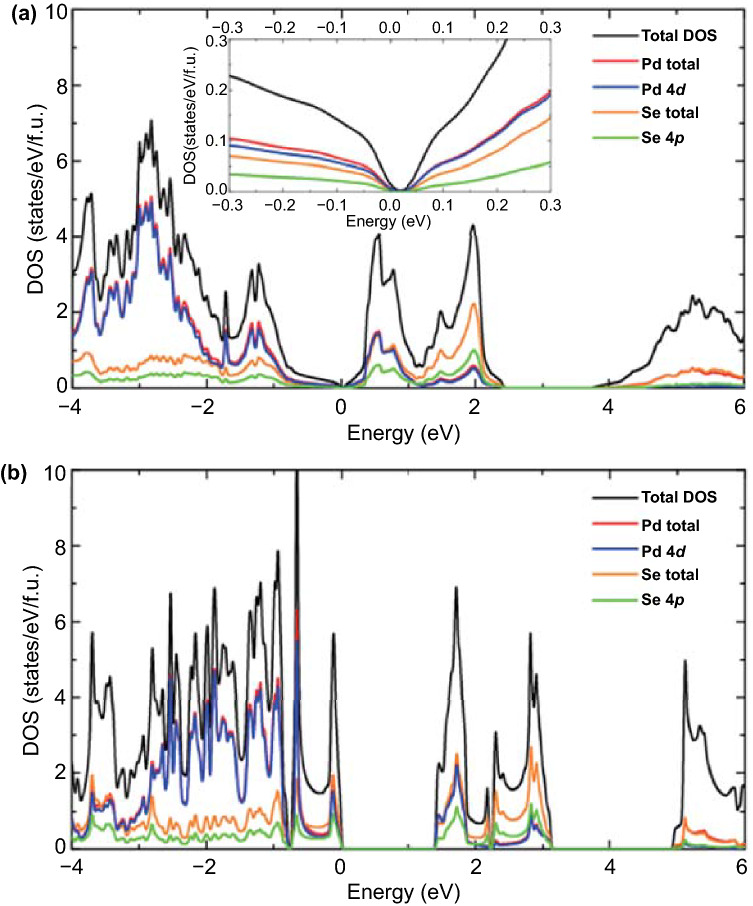
Calculated density of states of **a** bulk PdSe_2_ and **b** monolayer PdSe_2_. “DOS” denotes the density of states. Reproduced with permission from Ref. [27]. Copyright 2015, AIP Publishing LLC

Figure 3 depicts the electronic DOSs for both bulk and monolayer PdSe_2_ calculated in denser *k* meshes with values of 23 × 23 × 17 and 40 × 40 × 1, respectively [27]. In the inset of Fig. 3a, the bandgap of bulk PdSe_2_ is 0.03 eV, while that of monolayer PdSe_2_ is approximately 1.43 eV (Fig. 3b). These values are slightly higher than the bandgap values obtained through the traditional GGA-PBE functional, indicating an underestimation of the bandgap value. This uncertainty of the bandgap may be because PdSe_2_ has a high number of defects and in-plane anisotropic absorption properties.

In each layer, covalent bonding results in a distinct hybridization between the Pd 4d and Se 4p states. The projected DOSs show that the Pd 4d and Se 4p states contribute the most to the VBM and CBM, and the more substantial contribution of Pd 4d orbitals to the total DOSs increases at an energy below –1 eV [27].

### Vibrational Phonon Modes

Raman spectroscopy, which is a critical technique for 2D material characterization, was utilized to investigate the PdSe_2_ structure. In the Raman spectra of PdSe_2_, the peak position and intensity are shown to change anomalously with different numbers of PdSe_2_ layers, resulting from the electronic hybridization and strong interlayer coupling in the PdSe_2_ crystal [15].

**Fig. 4:**
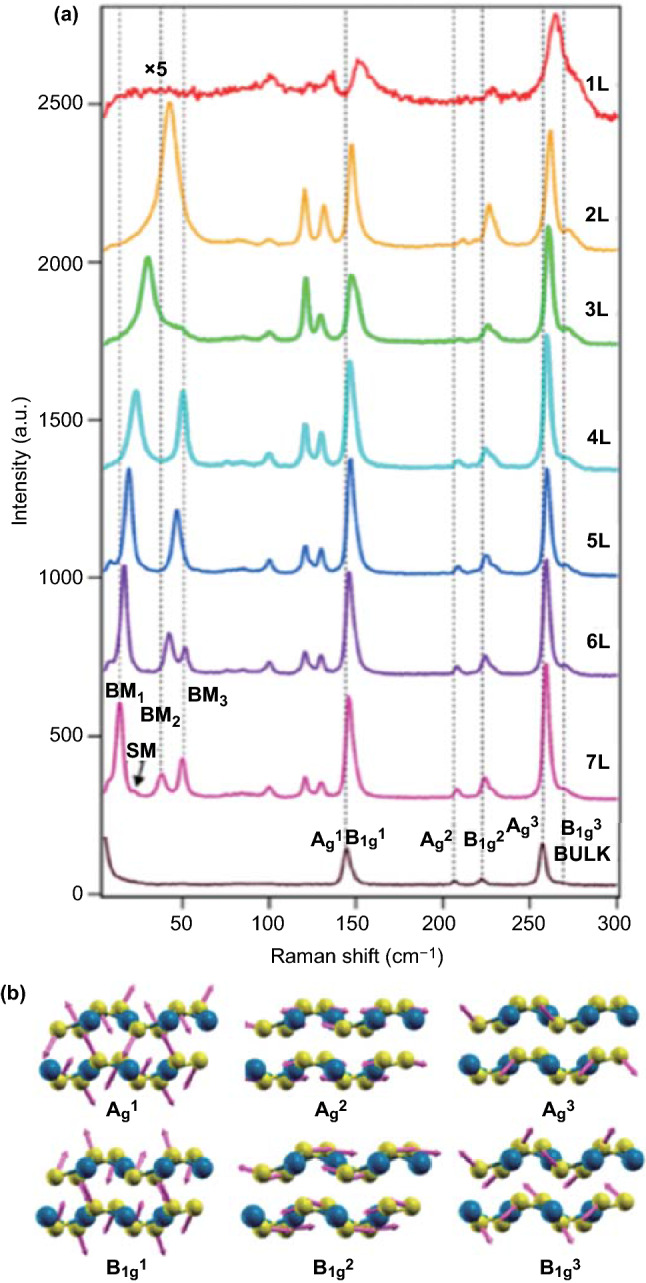
Vibrational properties of PdSe_2_. **a** Raman spectra of PdSe_2_ flakes of different layer number from monolayer to bulk. **b** Six major vibrational modes of PdSe_2_, which are labeled as A1 g, B1 1 g, A2 g, B2 1 g, A3 g, and B3 1 g. Reprinted with permission from Ref. [39]. Copyright 2020, American Chemistry Society

To provide a better understanding, Fig. 4a shows the Raman spectra of PdSe_2_ samples from monolayer to bulk, which demonstrates the evolution of the PdSe_2_ vibrational modes. There are four obvious peaks in the high-frequency (HF) Raman spectra region (100–300 cm^−1^), including six atomic vibrational modes [15]. The six peaks are at 144.3, 146.9, 206.7, 222.7, 257.8, and 268.6 cm^−1^, and the corresponding A_g_^1^, B_1g_^1^, A_g_^2^, B_1g_^2^, A_g_^3^, and B_1g_^3^ phonon modes of PdSe_2_ are marked with dotted lines in Fig. 4a. As the number of PdSe_2_ layers increases, the major peaks show a red shift, with the B_1g_^1^ peak changing the most and the A_g_^3^ peak changing the least. The main reasons for this are the in-plane lattice constant variations and the strong interlayer coupling of PdSe_2_, which causes abnormal shifts and a broad bandgap [15].

Figure 4b shows six atomic vibrational models, where the purple arrows represent the relative movements between the Pd and Se atoms. Among all the vibrational modes of PdSe_2_, the vibrations of Se–Se atoms are predominant. Indeed, the Se–Se bond presents a much stronger vibration intensity than that of the Pd–Se bond [39]. Moreover, there are three peaks in the low-wavenumber region (approximately at 101, 121, and 130 cm^−1^) owing to variations in the symmetry. As the number of PdSe_2_ layers decreases, the space group transforms from *Pbca* to *Pca*2_1_, leading to the emergence of the B_1g_^3^ mode and new peaks (268.6 cm^−1^) in few-layer PdSe_2_.

Low-frequency (LF) Raman spectroscopy (< 100 cm^−1^) was used to study the layer characteristics of PdSe_2_ further. As the two primary LF features, the breathing and shear modes pertain to the interlayer vibrational modes, and they depend on the relative motion perpendicular and parallel to the atomic layers, respectively. The breathing modes (BM_1_, BM_2_, and BM_3_) and shear modes (SM) are marked in Fig. 4a. For PdSe_2_, the intralayer covalent bonds along with the vibrational directions of adjacent atomic layers determine the intensities of the LF vibrational modes. Moreover, the interlayer vibrational modes display high intensities in few-layered PdSe_2_ flakes, even overtop the intralayer modes (HF features), which reflects the strong interlayer coupling of PdSe_2_. With the increase in the layer number of PdSe_2_, the LF Raman spectra exhibited a distinct red shift for the branches of the breathing modes. Such a shift was more pronounced than that of Raman peaks in the HF region. The full-width half-maximum (FWHM) of BM_1_ narrowed from 12 cm^−1^ (2 L) to 2.5 cm^−1^ (7 L) owing to the reduced phonon scattering rate in thicker PdSe_2_ flakes [18]. Thus, the number of PdSe_2_ layers can be precisely determined via Raman spectroscopy.

As mentioned above, PdSe_2_ presents relatively low symmetry owing to its puckered pentagonal structure, which exists in a few other TMDCs except PdS_2_. Thus, PdSe_2_ exhibits a unique anisotropy property, and the Raman scattering features of PdSe_2_ have been recently conducted to study the vibrational anisotropy [40].

### Polarization Properties

Compared with 2D TMDCs, PdSe_2_ possesses unique optoelectronic polarization properties because of anisotropy [16, 40], which is a great advantage for detecting polarized light. The PdSe_2_ has an appropriate bandgap (1.1 eV) and excellent optical absorption at the near-infrared range [40].

To date, PdSe_2_ remains the only choice for polarization investigation among the noble metal dichalcogenides. Indeed, the pentagonal PdS_2_ may possess the photoelectric properties analogous to the PdSe_2_. But 2D PdS_2_ investigation remains the theoretical calculation [13] and has yet been successfully prepared in experiments. This is probably because of the thermodynamic instability of marcasite PdS_2_ in the air [14]. Therefore, the application of PdSe_2_ exhibits high promise in the applications of optoelectronics and electronics.

**Fig. 5:**
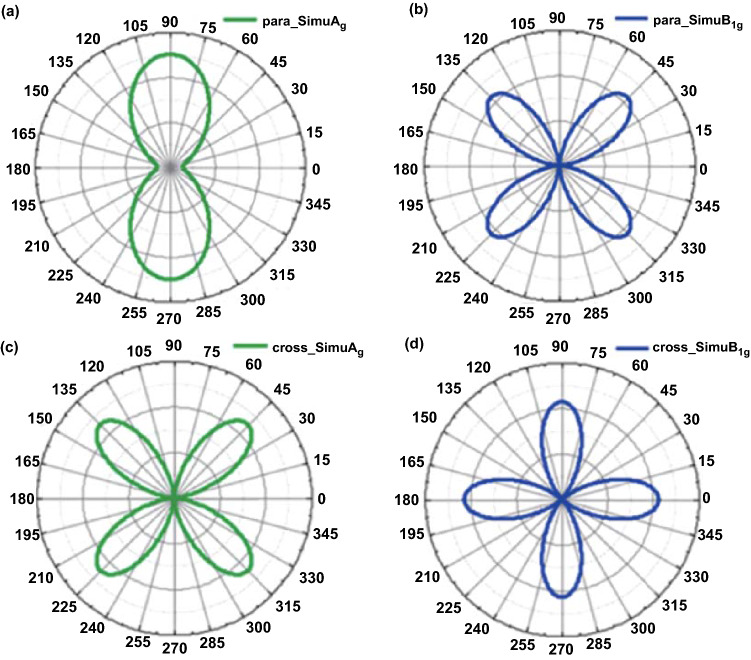
Polarization Raman intensities of PdSe_2_. The Raman intensity of A_g_ mode (**a**) and B_1g_ mode (**b**) under the parallel configuration with the simulation of the anisotropic modes. Raman intensity of A_g_ mode (**c**) and B_1g_ mode (**d**) under cross configuration of polarization Raman test. The layer number of PdSe_2_ is 3 for polarization Raman test. Reprinted with permission from Ref. [39]. Copyright 2020, American Chemistry Society

Polarization-resolved Raman measurements and theoretical calculations were employed to systematically investigate the anisotropic optical properties [39]. Figure 5a, b shows the Raman intensity simulations of the A_g_ and B_1g_ modes versus the polarization angle in 3 L PdSe_2_ under parallel polarization configuration. The A_g_ modes reveal a period of 180°, and the B_1g_ modes reveal a period of 90° in the parallel configuration.

Figure 5c, d presents the Raman intensity of both modes under parallel polarization configuration. Indeed, the A_g_ and B_1g_ modes both reveal a period of 90° under the cross configuration. The LF Raman peaks possess A_g_ or B_1g_ symmetry because the LF modes follow the group theory, similar to the HF modes, and the breathing modes and shear modes possess A_g_ and B_1g_ symmetry, respectively.

### Optical Absorption Properties

**Fig. 6:**
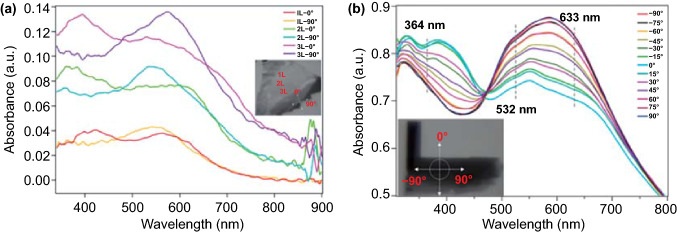
Polarized optical absorption of PdSe_2_. **a** Absorbance of 1–3 L PdSe_2_ along the *x*-axis (90°) and y-axis (0°). Inset: Optical micrograph of the PdSe_2_ flakes of different thicknesses. **b** Polarization-resolved absorption spectra of bulk PdSe_2_ within 300–800 nm spectra, with the measured angle from –90 to 90° in increments of 15°. Inset: Optical micrograph of the PdSe_2_ sample. Reprinted with permission from Ref. [39]. Copyright 2020, American Chemistry Society

The anisotropic features of PdSe_2_ can be verified based on its optical absorption. Figure 6a shows the optical absorbance of 1–3 L PdSe_2_ flakes at measurement angles of 0° and 90°, where an interesting orthogonal crossover is observed at around 470 nm [39]. Owing to the decrease in the bandgap, the increase in the number of PdSe_2_ layers leads to a slight red shift of the intersection point after 600 nm.

Figure 6b shows the variation in PdSe_2_ absorption with the polarization angle for a systematic investigation of the anisotropic characteristics. Almost all the absorption spectra of PdSe_2_ intersect at 472 nm when the polarization angle varies from –90° to 90°.

### Photoelectronic Properties

Based on the optical absorption of PdSe_2_, the photoresponse of 2D PdSe_2_ was investigated. The spatially resolved photocurrent mapping was collected for the few-layer PdSe_2_ devices [41]. Figure 4g shows a stable photocurrent of the device under 1060-nm illumination at two metal-PdSe_2_ junctions without any applied voltage.

**Fig. 7:**
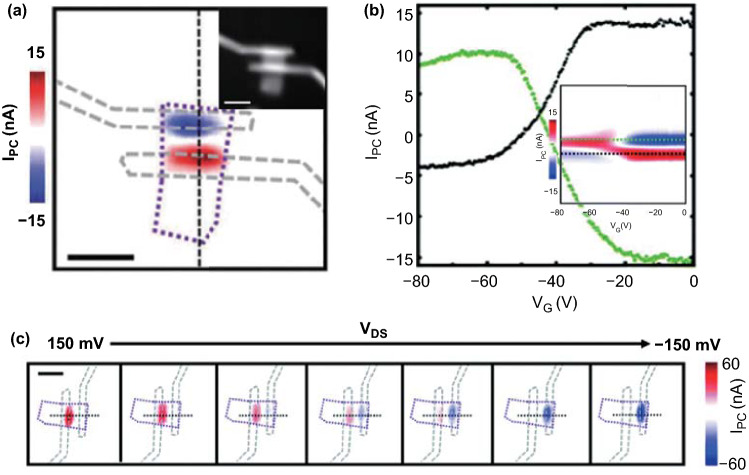
Photoelectric current mapping of PdSe_2_. **a** Scanning photocurrent images of the PdSe_2_ device under 1060-nm illumination with V_G_ = V_DS_ = 0 V, where the scale bar represents 5 μm. Inset: Reflection image of corresponding device with scale bar of 5 μm. **b** Photocurrents along the green and black dashed lines. Inset: Photocurrent signals as a function of gate voltage along the black dashed line in **a**. **c** Scanning photocurrent images of the PdSe_2_ device in **a** with V_DS_ from –150 to 150 mV, where the scale bar represents 5 μm. Reprinted with permission from Ref. [41]. Copyright 2019, Royal Society of Chemistry

To further study the photocurrent generation mechanisms, gate-dependent scanning photocurrent measurements were taken (Fig. 7a, b). Besides, the photocurrent could be tuned from positive to negative when regulating the drain–source voltage from 150 to -150 mV (Fig. 7c). The photocurrent mapping could be applied in the image sensing.

A strong photocurrent resonance peak emerges at 1060 nm, which may be due to an indirect optical transition. Due to the potential barriers created by the Fermi level alignment, a built-in electric field separates the photogenerated electron–hole pairs in the PdSe_2_ device [41].

### Thermoelectric Properties

Over the past decade, thermoelectric devices have attracted much attention because they can directly convert thermal energy into electrical energy. Because the bond saturation significantly enhances the thermal energy transport in 2D pentagonal materials, a unique feature is that PdSe_2_ possesses good thermoelectric properties. In particular, monolayer PdSe_2_ can be applied as a promising high-performance thermoelectric material in the future owing to its high Seebeck coefficient (> 200 μV K^−1^) [27]. For few-layer PdSe_2_, the energies of CB and VB were found to be convergent during a systematic investigation of its lattice structure and electronic properties, which indicates the significant thermoelectric properties of PdSe_2_ [42].

**Fig. 8:**
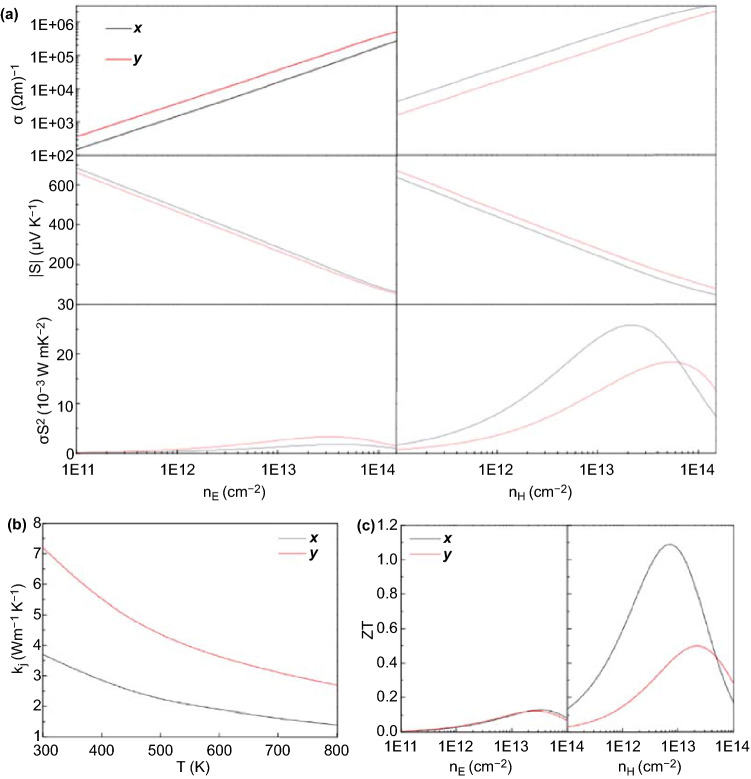
Thermoelectric properties of PdSe_2_. **a** Thermoelectric transport coefficients σ, S, and S^2^σ versus carrier concentration for PdSe_2_ with *n*-type (left) and *p*-type (right) doping at room temperature. **b** Lattice thermal conductivity of monolayer PdSe_2_ as a function of temperature. **c** Thermoelectric characteristics (ZT) of monolayer PdSe_2_ with *n*-type (left) and *p*-type doping (right) at room temperature. Adapted under the terms of the CC-BY Creative Commons Attribution 4.0 license (https://creativecommons.org/licenses/by/4.0/) from Ref. [138]. Copyright 2018, The Authors, published by Springer Nature

Figure 8a shows the electron transport coefficient of PdSe_2_ based on the constant relaxation time approximations of the Boltzmann theory [39]. Clearly, when the doped carrier concentration increased, the conductivity (σ) increased, while the Seebeck coefficient decreased. For monolayer PdSe_2_, the Seebeck coefficient can reach 660 μV K^−1^, which is comparable to that of some reported 2D materials [43]. The S for *p*-type doping is more asymmetric than that for *n*-type doping, and this provides the possibility for the design of transverse thermoelectric devices. Figure 8a proves that the power factor (PF) S^2^σ possesses distinct anisotropy, and this results from the large anisotropy of σ and S.

Figure 8b shows the calculation of the lattice thermal conductivity κ_l_ through the phonon Boltzmann transport equation and DFT. The lattice thermal conductivity of PdSe_2_ is much lower than that of monolayer MoS_2_ and GX_2_ [44], and it exhibits a large directional anisotropy. Figure 8c displays the relationship between the dimensionless figure of merit (ZT) value of the doped monolayer PdSe_2_ and the carrier concentration at room temperature.

The ZT value of monolayer PdSe_2_ is small and almost isotropic, while that for *p*-type doping is large and strongly anisotropic. Therefore, the high S, low σ, and high ZT values of monolayer PdSe_2_ at room temperature make PdSe_2_ suitable for thermoelectric devices.

### Phase Transformation Properties

Two-dimensional materials, especially TMDCs, can possess various properties via change in their phases, namely in terms of bonding and configurations, which can be exploited in other fields. For PdSe_2_, the interlayer interaction is relatively more reliable than the intralayer connection through covalent bonds, which facilitates the transition to other phases under different external parameters. The unique puckered pentagonal structure of PdSe_2_ possesses imperfect rotational symmetry, resulting in high defect sensitivity, particularly Se vacancies (V_Se_), which facilitates the occurrence of different phase transitions [45].

**Fig. 9:**
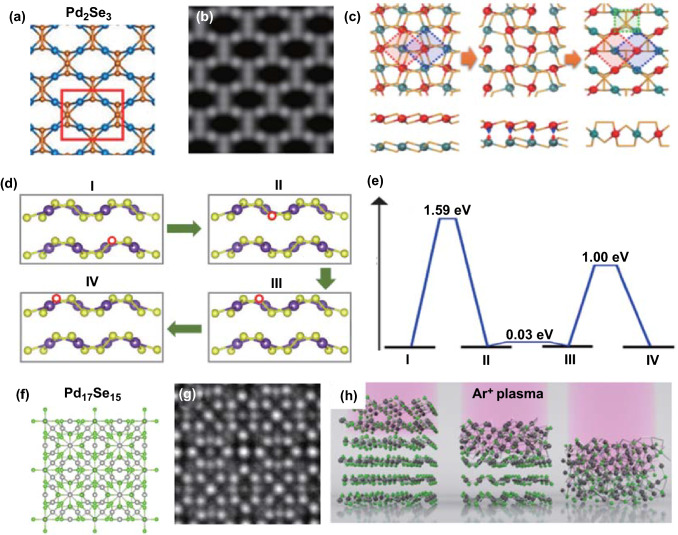
Atomic structure of different palladium selenide compounds. **a** Lattice structures and **b** corresponding simulated ADF-STEM image of monolayer Pd_2_Se_3_. Reprinted with permission from Ref. [47]. Copyright 2019, American Chemistry Society. **c** Schematic of reconstruction mechanism from bilayer PdSe_2_ to monolayer Pd_2_Se_3_, where the Se atoms are not presented. Reprinted with permission from Ref. [46]. Copyright 2017, American Physical Society. **d** Migration of V_Se_ configuration marked with the red circle in layered PdSe_2_. **e** Energy barriers of V_Se_ diffusions calculated between different configurations. Reprinted with permission from Ref. [45]. Copyright 2017, American Physical Society. **f** Lattice structures and **g** corresponding ADF-STEM image of Pd_17_Se_15_, where green and gray spheres represent Se atoms and Pd atoms, respectively. **h** Process diagram of Pd_17_Se_15_ formation from PdSe_2_ layer-by-layer through Ar plasma treatment. Reprinted with permission from Ref. [52]. Copyright 2019, American Chemistry Society

PdSe_2_ structure could transform into a Pd_2_Se_3_ structure (Fig. 9a) through V_Se_ [46]. From the STEM images, it was found that the preferred monolayer phase form exfoliated from bulk PdSe_2_ is not a PdSe_2_ structure. Through analysis of the quantitative STEM image intensity and DFT calculations, a new stable monolayer phase was determined to be Pd_2_Se_3_, which corresponds to the result from the experimental ADF-STEM image (Fig. 9b) [47].

The reconstruction of Pd_2_Se_3_ is due to the interlayer fusion mechanism, which results from the V_Se_ produced by electron radiation (Fig. 9c). According to the research results, the new Pd_2_Se_3_ phase exhibits physical stability and high cohesive energy, implying robust chemical bonding. Moreover, the Pd_2_Se_3_ monolayer is an excellent thermoelectric material with good electronic and optical properties [48].

Figure 9d shows the typical V_Se_ migration process in PdSe_2_ in four possible configurations labeled I, II, III, and IV. The red circle indicates the position of the V_Se_, which diffuses in the direction of the green arrow. The theoretically calculated energy barriers were presented for the corresponding V_Se_ diffusion (Fig. 9e). For configurations I to II and III to IV, the energy barrier of interlayer and intralayer V_Se_ diffusion is 1.59 and 1 eV, respectively. These barriers are lower than the corresponding energy barriers in MoS_2_. These V_Se_ migrations are facilitated by the stronger interlayer interaction and weaker intralayer bond strength of PdSe_2_. For configurations II to III, the energy barrier for intralayer V_Se_ diffusion is 0.03 eV owing to the Se–Se bonding [45].

Environmental energy input elevates the energy of PdSe_2_ and provides the activation energy for the formation of other Pd–Se compounds, viz. the phase transformation occurs. For example, the thermal annealing, plasma, and laser treatment have resulted in the phase transition of PdSe_2_. The typical external conditions are listed in Table 2 for the phase transition of PdSe_2_.

**Table 2:** The phase transition of PdSe_2_ under different conditions

Phase transition from starting phase	To final phase	Conditions	References
Pristine PdSe_2_ (2-4L)	Defective PdSe_2_ (Se vacancy)	400 °C annealing in vacuum for 10 s	[49]
Defective PdSe_2_	50% PdSe_2_ + 50% PdSe_2-*x*_ (*x* = 0–1)	400 °C in vacuum for 30 s	[49]
Partial PdSe_2-*x*_ (*x* = 0–1)	100% PdSe_2-*x*_	400 °C in vacuum for 30 s	[49]
PdSe_2-*x*_ (*x* = 0–1)	Pd_2_Se_3_ (striated; 1D channels)	400 °C heating in vacuum	[49]
Pd_2_Se_3_	PdSe	Vacuum annealing for Se loss	[49]
PdSe	Pd nanoparticles	Long vacuum annealing at 400 °C or heating at high temperatures (> 400 °C)	[49]
PdSe_2_	Pd_17_Se_15_	Ar plasma treatment	[52]
PdSe_2_	PdSe_2-*x*_ (*x* = 0–1)	Laser irradiation (60 µW)	[50]
PdSe_2_	Pd nanoparticles	Laser irradiation (600 µW)	[50]
Monoclinic PdSe_2_ (space group of I2/*a*)	Monoclinic PdSe_2_ (C2/m space group)	High pressure (4.5 GPa)	[40]
PdSe_2_	Hexagonal PdSe_2_ (P-3m1 space group)	High pressure (17.5 GPa)	[40]
Orthorhombic PdSe_2_	Ferroelastic PdSe_2_; Transition of layer stacking from *c* to a-axis orientation	Uniaxial compressive stress (0.6 GPa)	[33]

First, PdSe_2_ can be transformed to PdSe_2-*x*_ with vacuum annealing. According to the traditional bulk Pd–Se phase diagram [49], the Se loss induces the change in the Pd/Se ratio. Hence, the phase transition occurs after 30-s pulse annealing at 400 °C and the PdSe_2-*x*_ (*x* = 0–1) forms partially. Another 30-s pulse annealing completed the phase transition into Pd_2_Se_3_. The long-time annealing at 400 °C or heating at high temperature (> 400 °C) leads to excess Se loss and thinning of 2D materials and finally form pure Pd materials [49]. Indeed, Se loss occurs in other metal selenide upon thermal annealing. Second, high laser power can lead to Se loss and the formation of Pd nanoparticles [50]. Third, the high-pressure condition may induce the change of crystal structures [40] and layer stacking orientation [33].

Except for Pd_2_Se_3_, the Pd–Se binary phases include Pd_17_Se_15_, Pd_7_Se_4_, and Pd_4_Se. Through experiments, their metallic or superconducting characteristics have been displayed, and theoretical predictions have highlighted their topological quantum properties [51].

For instance, the Pd_17_Se_15_ phase has excellent stability with analogous chemical bonds to those of the PdSe_2_ phase [52]. Figure 9f, g shows the structure of the Pd_17_Se_15_ phase and the corresponding STEM images. The phase transition results from the V_Se_ in the PdSe_2_ crystal are due to Ar-plasma treatment (Fig. 9h). Moreover, the Raman spectra and STEM images indicate that the exposure time under Ar plasma irradiation affects the defects and degree of the phase transition in the PdSe_2_ crystal.

We now come to the introduction of synthesis strategies and posttreatment approaches.

## Synthesis Methods for Obtaining PdSe_2_

High-quality PdSe_2_ has been obtained via several reliable methods [17], which shows promise for exploration of its remarkable properties. In this section, we review the specific PdSe_2_ synthesis methods in terms of 3D bulk crystals and 2D thin films.

### Formation of 3D Bulk Crystals via Chemical Vapor Transport

The chemical vapor transport (CVT) method has been developed for the synthesis of most 3D bulk materials; it is an efficient method employed for laboratory synthesis and mass production. A common CVT reaction involves three processes: sublimation, transport, and deposition, and follows Le Chatelier’s principle in thermodynamics [53].

**Fig. 10:**
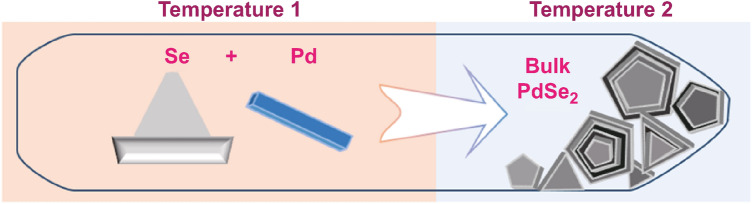
Scheme of the chemical vapor transport method for the bulk PdSe_2_ formation. The selenium power and Pd metal are sublimated in the left heating zone (Temperature 1) and cooling in the cold zone (Temperature 2) as bulk. The drawing was inspired by the literature [53]. The scheme was originally drawn by the authors in this review

The typical chemical vapor transport method has shown success in the growth of bulk PdSe_2_ crystals [54]. Herein, a stoichiometric ratio of high-purity Pd and Se powder was mixed as the source and placed into an ampoule reactor with mineralizers as the transporting agent (Fig. 10). The sealed reactor was then heated under a preset temperature gradient, where Temperature 1 is the temperature for the sublimation of Pd and Se and Temperature 2 is the temperature for PdSe_2_ deposition [54]. Generally, Temperature 1 is greater than Temperature 2 because the process of PdSe_2_ crystal formation is endothermic [53].

For example, Pd and Se powders (mixed in an atomic ratio of 1:6) were filled in a sealed evacuated quartz ampule, which was slowly heated to 850 °C and maintained for 50 h. After the synthesis was completed, the quartz ampule was gradually cooled to 450 °C at a rate of 3 °C h^−1^ and finally naturally cooled to room temperature [53]. Eventually, shiny single PdSe_2_ crystals were obtained on millimeter-grade paper.

### Developing 2D Thin Tilm via Exfoliation

As devices with smaller sizes and higher performance are desired in the development of electronics, the growth of high-quality ultrathin 2D materials has become increasingly crucial. Thus, mechanical exfoliation and chemical vapor deposition (CVD) techniques are widely employed to produce layered PdSe_2_ thin films.

**Fig. 11:**
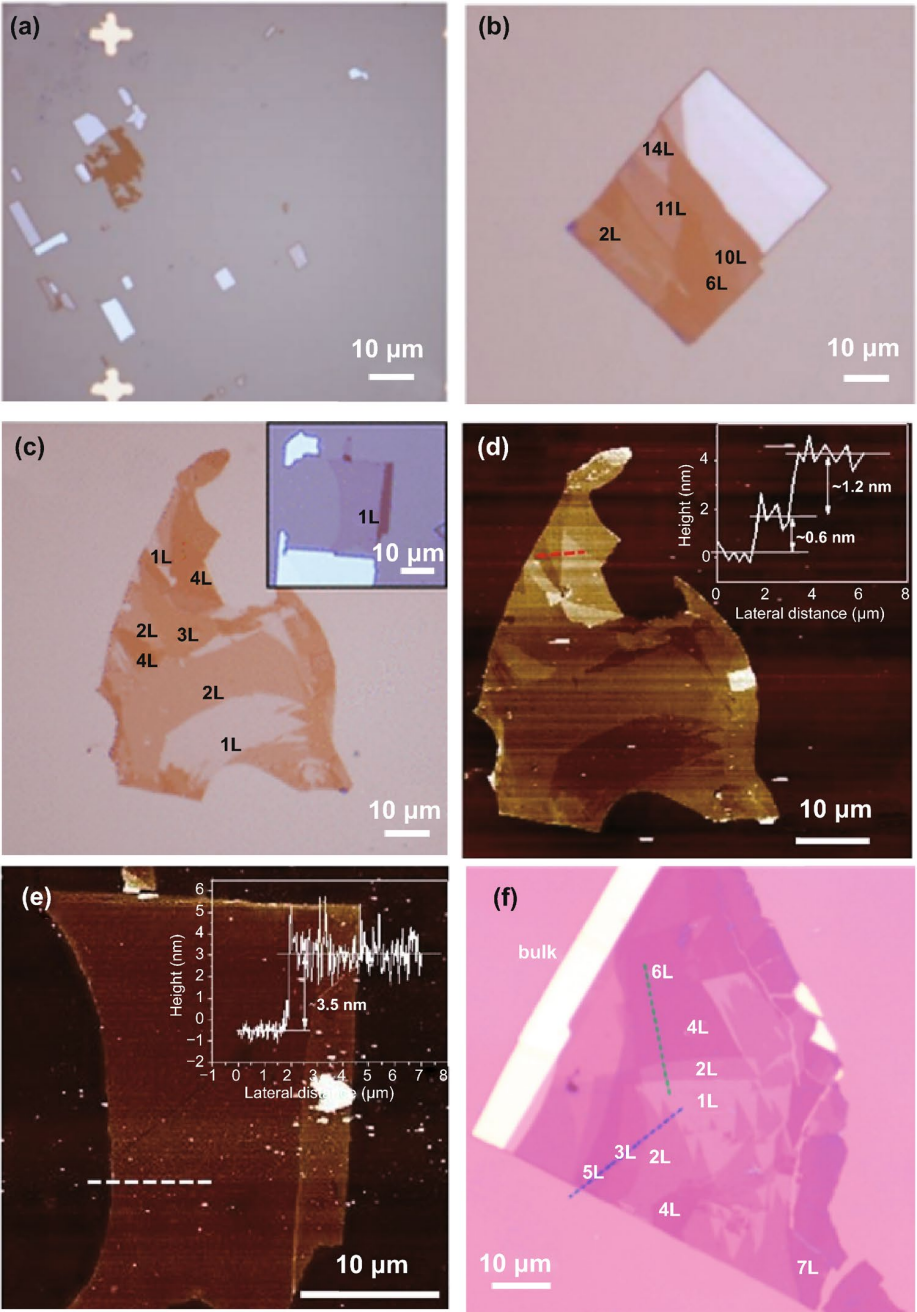
Mechanically exfoliated PdSe_2_ flakes. **a** Optical micrographs of exfoliated PdSe_2_ nanosheets on the substrate with lithographed metal marks. **b, c** Optical micrographs of PdSe_2_ flakes at different regions. **d, e** Atomic force microscopy images of PdSe_2_ samples from the region at the panel **c** and its inset. Reprinted with permission from Ref. [15]. Copyright 2017, American Chemistry Society. **f** Optical microscopy images of the PdSe_2_ flakes with different layers. Reprinted with permission from Ref. [39]. Copyright 2020, American Chemistry Society

After the synthesis of bulk PdSe_2_ crystals, atomic PdSe_2_ thin flakes could be easily obtained using the mechanical exfoliation method [15]. PdSe_2_ flakes with different layers were transformed onto the Si/SiO_2_ substrate (Fig. 11). The exfoliated PdSe_2_ samples were then applied to different electronic devices.

The exfoliated PdSe_2_ flakes have high crystallinity (Fig. 11) and intrinsic properties, which are beneficial for fabrication of individual devices [39]. The mechanical exfoliation method enables facile fabrication of the vdWHs [54]. However, the lack of large-area uniformity and layer-number controllability limits the applicability of the mechanical exfoliation method; moreover, the method is difficult to use for industrial production.

The typical features are compared in Table 3 for the synthesis approaches of 3D bulk, nanosheets, and 2D films of PdSe_2_.

**Table 3:**
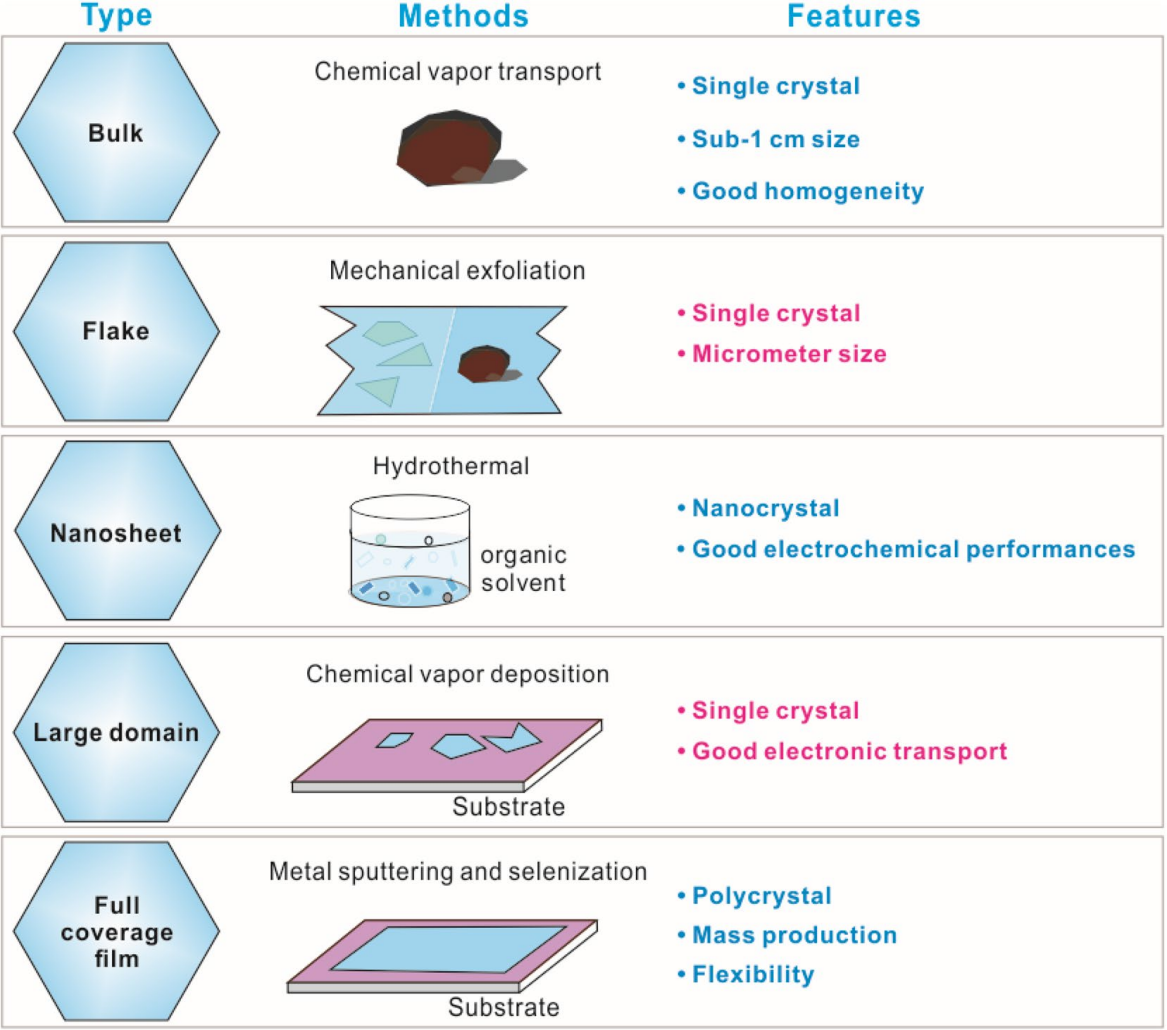
Different types of PdSe_2_ from the various synthesis approaches. These scheme were drawn by the authors, which were inspired by the literatures, i.e., bulk [17], flake [42], nanosheet [137], large domain [27] and 2D film [138]

Recently, the Au-assisted exfoliation method has shown success in the separation of monolayer 2D materials over a centimeter size [55]. In brief, the Au film is first deposited onto a target substrate [56]. Then, the tape with exfoliated 2D material is stuck onto the Au surface. Upon the pressing over the sample, the strong interaction forms between Au and 2D material. Eventually, monolayer or few-layer 2D materials remain over the Au surface after peeling off the tape. Here, the interlayer interaction in TMDCs can be overcome by the interaction between Au film and 2D materials [57]. The strong van der Waals interaction between Au and the uppermost two-dimensional layered transition-metal chalcogenide promotes the exfoliation of the single layer, which leaves large-area single-layer domain on the Au surface. For example, Au-assisted exfoliation has produced large MoS_2_ domains, i.e., 40 times greater than that produced by the tape-assisted exfoliation [57].

The Au-assisted exfoliation has become a universal approach for obtaining millimeter-sized 2D materials including PtSe_2_, PtTe_2_, and PdTe_2_ [58]. It may apply to the exfoliation of PdSe_2_ over a large size soon, which may accelerate the fabrication of electronic device arrays due to the large effective film area. The 2D materials over Au film by Au-assisted exfoliation can be applied in electrochemistry and photocatalyst [55].

Most 2D materials with large-area uniformity and high crystallinity can be synthesized via the CVD method or thermal selenization/sulfurization treatment [59]. Several approaches have been used to grow homogeneous PdSe_2_ thin films, with satisfactory results being obtained. We now discuss thermal deposition approaches for synthesizing PdSe_2_ films.

### Chemical Vapor Deposition from the PdCl_2_ and Se Reaction

**Fig. 12:**
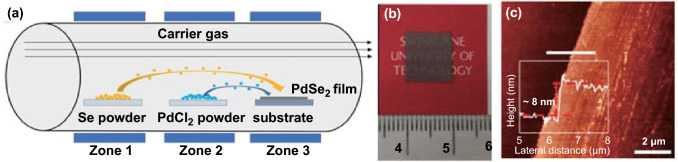
**a** Schematic of PdSe_2_ synthesis process using CVD method. **b, c** Photograph and AFM height profile of a prepared multilayer PdSe_2_ film. Reprinted with permission from Ref. [60]. Copyright 2020, American Chemical Society

A chemical vapor deposition strategy was developed by employing Pd-containing precursors and Se powders for synthesizing the PdSe_2_ films. Here, PdCl_2_ powder was selected as precursors [60]. A schematic of the CVD process with a three-zone tube furnace is shown in Fig. 12a.

Here, Se powder was placed in Zone 1 at a temperature of 250 °C, and PdCl_2_ powder was placed in Zone 2 at a temperature of 500 °C. Then, Se and Pd precursors were transported by Ar/H_2_ to Zone 3, and the temperature was maintained at 600 °C, at which the polycrystalline PdSe_2_ films were synthesized continuously on the substrate. Figure 12b shows a photograph of the as-grown PdSe_2_ film with high uniformity. The AFM image and height profile of the PdSe_2_ films were characterized (Fig. 12c) with a thickness of ∼8 nm, corresponding to 20 layers of PdSe_2_ [15].

Because of the high melting point of the Pd metal precursor, the molten salt-assisted method can be utilized for the growth of PdSe_2_ flakes, which can be synthesized at a lower temperature over a large domain [61]. The ambient pressure chemical vapor deposition (APCVD) method can be used with the assistance of salt powder, such as NaCl, where the Pd metal precursor is replaced by high-purity PdCl_2_ powder. Au foils were placed above the mixture and heated at 850–900 °C at 85 sccm Ar and 15 sccm H_2_ flows for 10–15 min. Interestingly, the length/width ratio of the PdSe_2_ flakes increased markedly during the synthesis. PdSe_2_ flakes were obtained with growth times of 20 and 35 min, respectively. The PdSe_2_ flakes on Au foil exhibited a ribbon-like shape, which was rarely the case on the amorphous oxide substrates. Hence, the synthesis of PdSe_2_ may depend on its anisotropic structure and orthorhombic symmetry.

### Chemical Vapor Deposition Reaction by the Sublimated Pd and Se

**Fig. 13:**
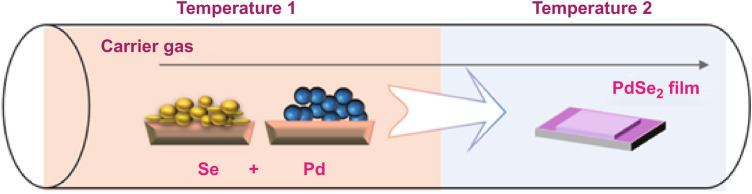
The chemical vapor deposition synthesis of the PdSe_2_ film. The Se power and Pd metal are sublimated in temperature 1 and deposited at temperature 2 for 2D film synthesis. The concept was inspired by Ref. [62]. The scheme is originally drawn by the authors of the review

A CVD approach has been developed with the reaction of sublimated Pd and Se for growing few-layer PdSe_2_ flakes with high crystallinity [62]. In the setup for the synthesis of PdSe_2_ crystals, the Se powder was placed in a separate quartz tube zone wrapped with a heating belt at 350 °C, while Pd powder was located in the center of the furnace at 800 °C, with an Ar flow of 50–150 sccm for 10–20 min. Meanwhile, the substrate was placed in the downstream zone outside the heating zone at 480–600 °C. The scheme of the growth method is presented in Fig. 13.

Notably, the PdSe_2_ flakes had various thicknesses, sizes, and shapes when the substrates were synthesized at different temperatures. For example, square-like flakes grown at 600 °C are thicker and larger than the heart-like flakes grown at a temperature of 500 °C.

Chemical vapor deposition has been employed for synthesizing large-area PdSe_2_ films [16], single-crystal domains [63], nanowires [48], and ribbons [64]. Wafer-scale single-crystal PdSe_2_ may be necessary for integrated circuit applications.

### Selenization of Pd Film

A simple selenization method leads to the synthesis of noble metal diselenide films [65]. The synthesis of PdSe_2_ films by direct selenization and the thickness of PdSe_2_ can be well controlled by varying the thickness of the deposited Pd layer [62]. The Pd layer deposited on the substrate via magnetron sputtering was placed in the center zone of the tube furnace at 480 °C, while the high-purity Se powder (99.99%) was placed in the upstream zone at 220 °C under a 60-sccm Ar flow for 90 min. The selenization strategy could enable the wafer-scale growth of PdSe_2_, such as in the form of a 2-inch PdSe_2_ film over a Si wafer [62]. The Raman mapping of the PdSe_2_ film proves that the PdSe_2_ film possesses good uniformity.

The structure–property relationship is listed in Table 4. The advantages and disadvantages are compared for different synthesis approaches for obtaining PdSe_2_. Future opportunities lie in the synthesis of monolayer single-crystal PdSe_2_ full film over a wafer scale (yet shown).

**Table 4:** Comparison of the types of PdSe_2_ from different methods

Methods	Structure quality	Types	Thickness (nm)	Average domain size (µm)	Mobility (cm^2^ V^−1^ s^−1^)	Advantages	Disadvantages	References
Mechanical exfoliation	Single crystal	Flake	0.6 – 2.4	30	158	High crystalline quality; micrometer-scale grain size;	Not compatible with mass production; irregular shape; inhomogeneity in thickness	[15]
Mechanical exfoliation	Single crystal		6.8 – 116	5 – 10	130 (at 300 K) and 520 (at 77 K)	High quality from CVT-derived PdSe_2_ bulk material	Large time cost; large human resource cost for repeating the exfoliation by human hands;	[139]
CVD from PdCl_2_	Nanocrystal	Film	8	From 0.01 to 0.1	n.a	Centimeter-scale film growth; industrial mass production potential;	Small grain size	[60]
CVD from Pd powder over Si/SiO_2_ substrate	Nanocrystal	Film	3 – 12	From 3 to 5	294	Large-scale production promise	Small grain size	[16]
CVD over sapphire and mica substrate	Single crystal	Square domain	1.2 – 2.4 (2 L and 4 L)	5 – 10 (sapphire); 5 – 10 (mica);	n.a	High crystalline quality;	Fragile sapphire substrate; Not tolerant with fast cooling after CVD growth	[16]
CVD over Au substrate	Single crystal	Large domain	1.2 (bilayer)	200 µm long and 2 µm wide strip;	n.a	Large lateral grain size;	Expensive Au substrate	[16]
Metal film plus selenization	Nanocrystal	Film	1.2 – 20	From 0.03 to 0.05	n.a	Simple process; wafer-scale production	Small grain size; nanocrystalline; low crystalline quality;	[62]
Pd dimer and selenization over epitaxial substrates	Nanocrystal	Flake	5	From 0.005 to 0.01	n.a	Large single crystal	Small grain size; small-scale; irregular shape	[22]
Thinning of PdSe_2_ flakes by etching	Single crystal	Flake	n.a	3	n.a	Regulating the layer number of PdSe_2_; modulating the physical properties of PdSe_2_;	The grain size depending on the pristine PdSe_2_ material;	[66]
Ideal CVD (to be investigated)	Single crystal	Film	0.6 (monolayer)	Beyond 100 µm [139]	> 1000 (theoretical limit) [17, 19, 27]	Mass production potential; wafer-scale production; High-quality single crystal	n.a	To be announced

### Direct van der Waals Epitaxial Growth of PdSe_2_ on Graphene

**Fig. 14:**
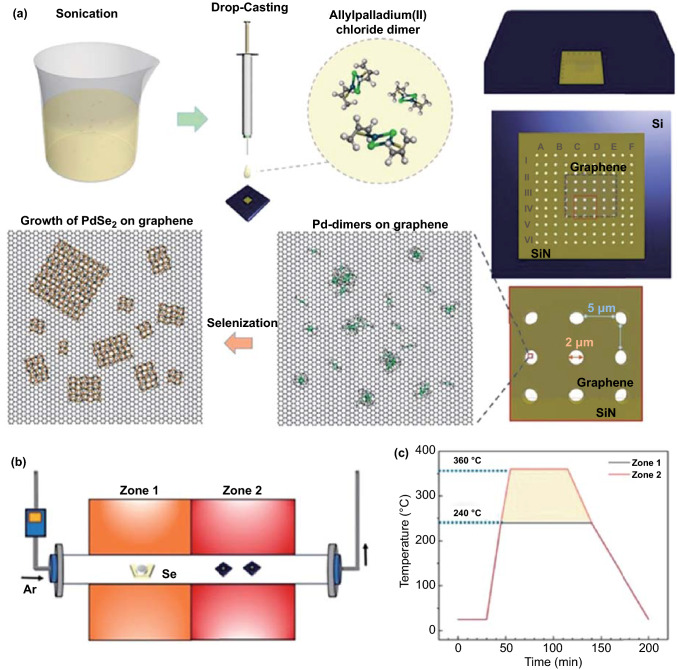
Growth of PdSe_2_ over graphene with selenization of Pd dimers as precursors. **a** Schematic of protocol for PdSe_2_ synthesis. **b** Schematic of two-zone horizontal furnace for thermal treatment under Se-rich atmosphere. **c** Temperature distribution along with tube furnace. Reprinted with permission from Ref. [22]. Copyright 2020, American Chemical Society

The PdSe_2_ has been deposited over the support of graphene or MoS_2_ in an epitaxial growth fashion [22]. The precursor of Pd containing organic molecules has been employed for the formation of PdSe_2_. Figure 14a illustrates a schematic of the experimental process. The van der Waals heterostructure of PgSe_2_/graphene can be directly grown with this method.

Graphene was suspended on top of the observation membrane by drop-casting the same volume of dispersion onto a TEM grid. The graphene was transferred onto a 0.50 × 0.50 mm^2^ SiN_x_ membrane, which has 2-μm vacuum pinholes spaced 5 μm apart.

Figure 14b shows the CVD system for the selenization of PdSe_2_. The two-zone furnace was compiled with the temperature profile for Zone 1 at 240 °C and Zone 2 at 360 °C (Fig. 14c). This research presents a direct method for the growth of vdWHs at the nanoscale and atomic level and an innovative strategy for the synthesis of 2D materials through predetermined nucleation.

### Layer-by-layer Thinning by the Oxygen Plasma

**Fig. 15:**
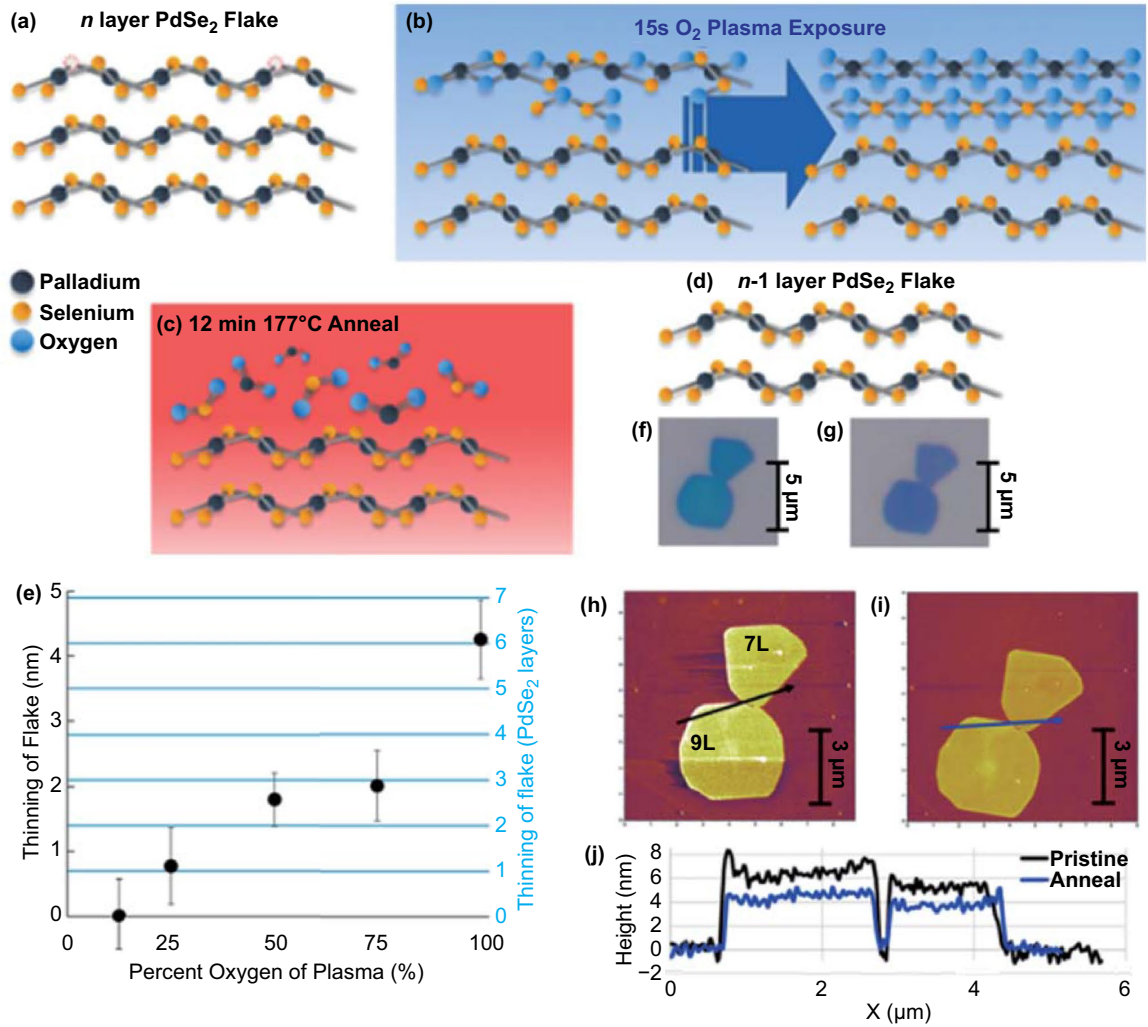
Thinning of PdSe_2_ layers with plasma treatment. **a** Pristine PdSe_2_ flakes. **b** Oxygen plasma etching. **c** Thermal annealing. **d** Resultant PdSe_2_ after layer thinning. **e** Etching of layers versus the oxygen percentage in the plasma. Optical micrographs of PdSe_2_ **f** before plasma etching and **g** after plasma thinning. Atomic force microscope micrographs of PdSe_2_ flakes before **h** and after **i** layer thinning. **j** Height profiles from two lines extracted from panel **h** and panel **i**. Reprinted with permission from Ref. [66]. Copyright 2020, American Chemical Society

Precise layer control of PdSe_2_ samples plays an important role in tuning of the bandgap of PdSe_2_. A layer-by-layer thinning strategy has been employed for etching an *n*-layered PdSe_2_ flake to the (*n* − 1) layered flake (Fig. 15). Precise layer thinning [66] has been depicted by selective oxidation via oxygen plasma and sublimation through thermal annealing (Fig. 15a-d).

To investigate the etching method, the PdSe_2_ flakes were exposed to plasma with different O_2_/Ar ratios [66]. Figure 15e shows the variation in the thickness of the PdSe_2_ flakes after etching. The correlation between the thickness and number of layers employs an empirical value of 0.7 nm per PdSe_2_ layer [15]. Figure 15f shows an optical micrograph of two pristine PdSe_2_ flakes with seven and nine layers, respectively. Figure 15g shows the same regions after the plasma etch cycle. The color of the PdSe_2_ species changes subtly from blue to light purple, which indicates a decrease in the PdSe_2_ film thickness.

The AFM images of the corresponding PdSe_2_ flakes (Fig. 15h, i) provide line-scanning information (Fig. 15j). Here, 2-nm PdSe_2_ (ca. 3 layers) was etched after oxidation and sublimation upon O_2_ plasma treatment. Therefore, plasma etching and surface curing may shed light upon the bandgap regulation of 2D materials over a large area.

The posttreatment of PdSe_2_ could modify the structure and properties of the pristine material. First, mild plasma exposure to PdSe_2_ could lead to layer-by-layer plasma etching to regulate the thickness [66]. The ozone treatment [67] of PdSe_2_ could enhance the chemical sensitivity owing to the weak oxidation. Electron irradiation can modify conductivity performance [68]. The phase transformation of PdSe_2_ leads to a sub-1-nm channel by thermal treatment [49] and the Pd_2_Se_3_ phase by interlayer fusion [46].

## Roles in Electronic Devices

As mentioned above, because of the strong interlayer interactions resulting from the almost fully occupied *d*-orbital and tunable properties, which depend on the number of layers, PdSe_2_ shows potential as a 2D material applicable for use in electronic devices.

### Electrical Contacts for PdSe_2_ Devices

Prior to fabrication of an electric device, a metal/PdSe_2_ contact is essential for optimizing the electrical performance of transistors, photodetectors, and integrated circuits. At the interface of metal/semiconductor contact, the transport properties of charge carriers are determined by the Schottky height, tunneling energy barrier, orbital overlapping percentage, as well as the geometry of the interface.

**Fig. 16:**
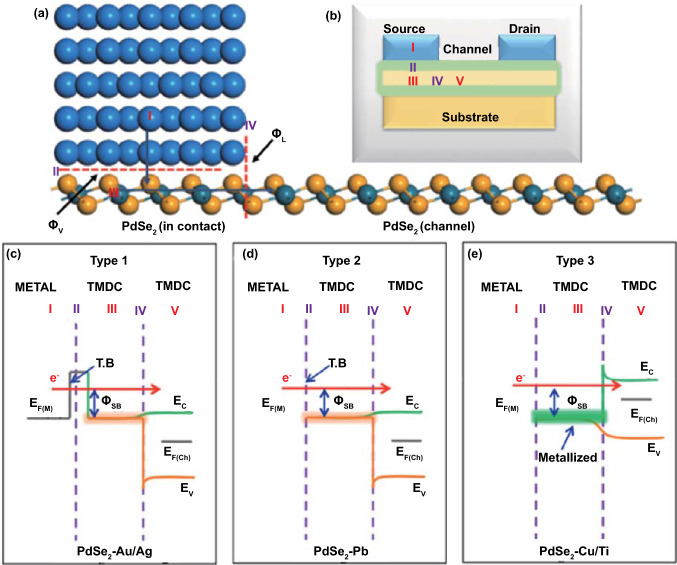
Metal/PdSe_2_ contact, transistor configuration, and their band alignment. **a** Atomic configuration of PdSe_2_-metal contact. The pathway of electron flows is coordinated from the metal electrode to the metal/PdSe_2_ interface and then to the PdSe_2_ channel. **b** Schematic of typical PdSe_2_ field-effect transistor. The labeling is identical for the five panels of I, II, III, to IV and V. Energy band alignment of different PdSe_2_–metal contacts based on tunneling evaluation and Schottky barriers with **c** weak bonding by Au/Ag, **d** medium bonding with Pb, and **e** strong bonding by Cu/Ti interface. “T.B.” denotes the tunneling barrier. Reprinted with permission from Ref. [69]. Copyright 2020, American Chemical Society

Theoretical calculations using the DFT approach were employed to compare the metal/PdSe_2_ contact performances by tuning the metal types such as Au, Ag, Pb, Cu, and Ti, as well as semimetallic graphene. The efficiency of charge transfer at the PdSe_2_–metal interface was examined for energy barrier evaluation [69]. Figure 16a shows the prototype of a PdSe_2_–metal contact with a carrier flowing from the metal electrode to the PdSe_2_ channel through the pathway (I → II → III → IV → V). In a typical PdSe_2_ FET (Fig. 16b), carriers diffuse from the metal to the layered PdSe_2_ and encounter a tunneling barrier, which depends on the binding strength at the interface of the PdSe_2_-metal contact.

The PdSe_2_–metal contacts, i.e., with Au or Ag electrodes, are demonstrated with their energy band alignment based on the binding energy owing to the Schottky barriers (Fig. 16c). First, Au/PdSe_2_ was preferred via compression of the Schottky barrier height. Meanwhile, the Ag electrodes led to an improved orbital overlap with PdSe_2_. A vertical Schottky barrier appears at the interface (II) in the vertical direction, while a lateral Schottky barrier occurs at the interface (IV) between the heterojunction and the PdSe_2_ channel region. Second, the Pb/PdSe_2_ contact has a low tunneling potential with a Schottky barrier height of 0.67 eV (Fig. 16d). Third, Cu/PdSe_2_ does not form a tunneling interface (Fig. 16e) but has a Schottky height of 0.58 eV.

Eventually, the graphene/PdSe_2_ contact has emerged as a proof of concept with regard to vdWHs. A Schottky barrier height of 0.22 eV is preferred for electron transport—that is, *n*-type charge carrier conductance [69]. Indeed, electrons are transferred from the interface to the PdSe_2_ side with a band bending of − 0.94. The weak van der Waals interactions between graphene and PdSe_2_ render a quasi-Ohmic contact without energy transfer between the two surfaces. That is, the intrinsic transport properties of PdSe_2_ are maintained. Analogous to the contact behavior of other 2D materials, one can fabricate high-performance optoelectronic devices.

**Fig. 17:**
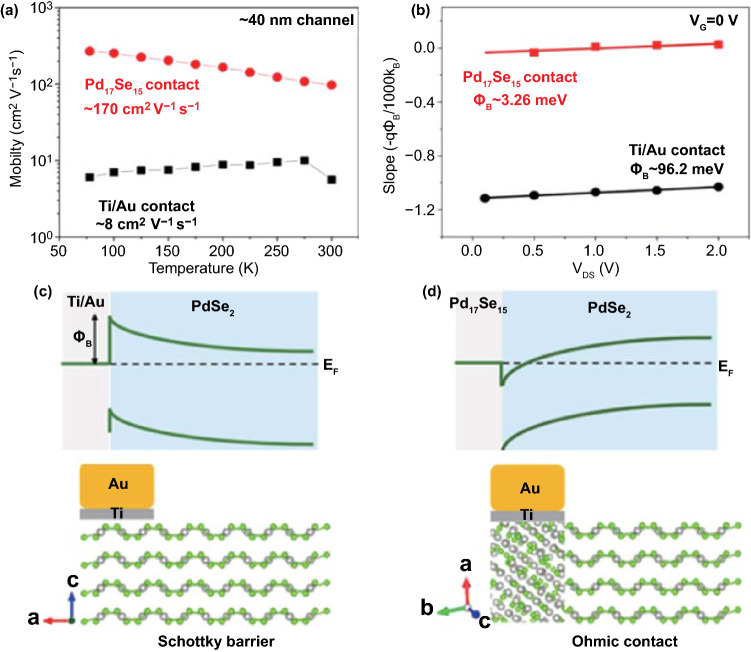
Metal/PdSe_2_ contact for regulating the electronic transports. **a** Comparison of temperature-dependent mobility of the PdSe_2_ channel with Ti/Au and Pd_17_Se_15_ contacts, respectively. **b** Comparison of V_DS_-dependent Schottky barrier height of PdSe_2_ devices with Ti/Au and Pd_17_Se_15_ contacts, respectively. Schematic of **c** Ti/Au contact and **d** Pd_17_Se_15_ contact. Reprinted with permission from Ref. [52]. Copyright 2019, American Chemistry Society

The stability and metallicity of the Pd_17_Se_15_ phase make it an ideal buffering material between the metal and PdSe_2_. The low lattice mismatch between both palladium selenides guarantees quasi-Ohmic conductance behavior, which suppresses the Schottky barrier height. In contrast, PdSe_2_ devices with Pd_17_Se_15_ contacts performed better than those with Ti/Au contacts [52]. Figure 17a shows the temperature-dependent mobility of PdSe_2_ devices with Pd_17_Se_15_ contacts and Ti/Au contacts, which are approximately 170 and 8 cm^2^ V^−1^ s^−1^, respectively.

Figure 17b shows the relationship between the slope and different drain voltages at zero gate voltage. The Schottky barrier height Ф_B_ of the Ti/Au contact device (96.2 meV) is greater than that of the Pd_17_Se_15_ contact device (3.26 meV). Figure 17c, d illustrates the electrode contact of the PdSe_2_ devices from the Schottky barrier of Ti/Au contacts to the Ohmic contact of Pd_17_Se_15_ contacts. Thus, the contact resistance of the PdSe_2_ device decreases and has the potential to become closer to the quantum limit.

Future opportunities may remain in developing the electrical contacts of PdSe_2_ with other palladium selenides. Indeed, the PdSe_2-*x*_ phases with different stoichiometric ratio may arouse different contact behaviors when stacking vertically with PdSe_2_ or stitching together laterally. The PdSe_2-*x*_/PdSe_2_ contact could be either Ohmic or Schottky typed, which require the optimization of researchers. The Schottky type contact could be utilized in the rectifier device. The Ohmic contact facilitates the electronic transport performances such as charge carrier mobility. The phase-engineering method proves that new crystalline phases of anisotropic 2D materials can be induced by defects. These new PdSe_2-*x*_ compounds may have different stoichiometries, which broadens the choices of materials for electrical contacts.

After understanding the metal/PdSe_2_ contact, we now come to the discussion of electronic transport performances in field-effect transistors.

### PdSe_2_ Field-effect Transistors

The field-effect transistors are one of the most significant devices in semiconductor electronics, and FETs based on 2D materials have shown superior performance to those based on traditional semiconductors. Moreover, usage of 2D materials provides new opportunities and effective approaches regarding FETs, with a high on/off ratio, high carrier mobility, and excellent stability. The layer-dependent properties of TMDCs are important for the design of FETs for diverse functionalized devices [30]. In this section, PdSe_2_ FETs and efficient methods to improve their performance are introduced.

**Fig. 18:**
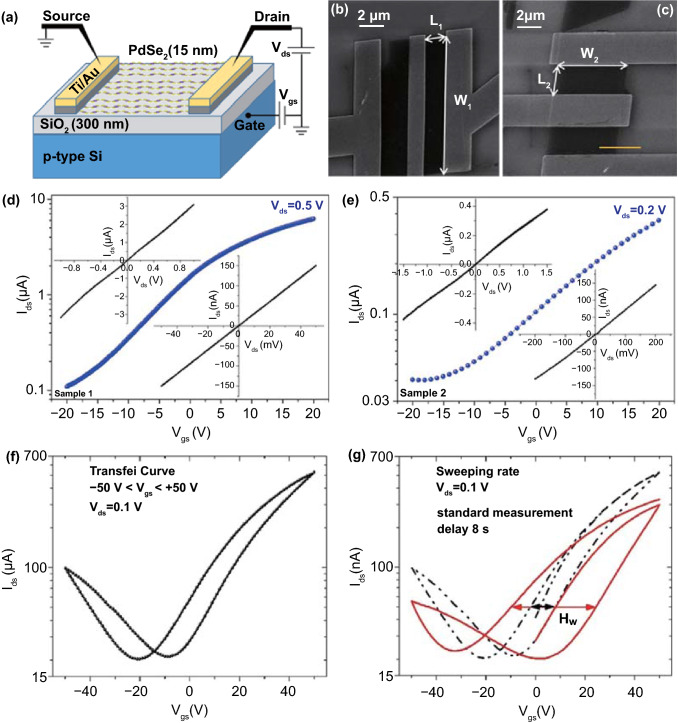
Demonstration and performance of PdSe_2_ FET. **a** Schematic of PdSe_2_ field-effect transistor and electrical measurements. **b, c** SEM micrographs of two devices with source and drain electrodes fabricated on 15-nm-thick PdSe_2_ flakes. Channel length and width vary in both transistors. **d, e** Transfer characteristics of PdSe_2_ FETs corresponding to **b, c**. Insets are the drain current versus voltage, i. e., the output characteristics of the PdSe_2_ transistor at the high bias voltage (top left) and low bias voltage (bottom right). **f** The transfer curves of the PdSe_2_ FETs measured at vacuum conditions of 10^–6^ mbar. **g** Comparison of the transfer curves in panel **f** with the curves measured after a delay of 8 s relative to the standard measurement time in panel **f**. The Hw denotes the hysteresis width. Reprinted with permission from Ref.[74]. Copyright 2019, Elsevier Ltd

PdSe_2_ has proven to be a successful channel material for FETs. PdSe_2_ transistors have achieved high mobility with tunable ambipolar characteristics [70]. Figure 18a shows an experimental setup to measure the PdSe_2_ FET characteristics with *p*-type Si as a universal back-gate electrode, and Fig. 18b, c shows SEM images of the two as-fabricated PdSe_2_ FET samples.

The electronic performances of the PdSe_2_ FETs are depicted in the output and transfer characteristics (Fig. 18d, e). In the output curves, the PdSe_2_ FET exhibited a linear correlation between the voltage and current (insets of Fig. 18d, e). Such a linear drain current–voltage dependence indicates quasi-Ohmic contact, i.e., suppression of the Schottky barrier between PdSe_2_ and the electrodes. The electrons dominate the primary charge carriers of the FET at a positive gate voltage and a small negative gate voltage (Fig. 18f). In contrast, the holes become the leading charge carriers of the FET at a large negative gate voltage.

The air stability of PdSe_2_ guarantees the lifetime of its transistor-based sensor applications in a wet environment. For example, the 2D material-based transistors have been embedded in a microfluidic chip for microRNA detection and screening [71]. The chemical sensors of 2D materials have demonstrated superior performances.

The slight hysteresis of the transfer curve could result from the effect of slow trap states and the surface adsorbates from the lithography-based fabrication process [17]. Remarkably, the ambipolar behavior of the PdSe_2_ FET could be regulated through a biased sweep.

The hysteresis width can be periodically evaluated by continuously measuring several transfer curves. These two transfer curves of the PdSe_2_ FET were measured in succession (Fig. 18g). The first curve in black was employed as a reference curve, and the second curve in red was collected at a delay of 8 s after the first signal capture. Therefore, the hysteresis is elevated with the increased sweeping time of the gate voltage.

The transport properties of PdSe_2_ FETs can be regulated by employing vacuum annealing, charge doping, electrical stress, pressure, and electron irradiation [17]. Through reduction of both the pressure and electron irradiation, hysteresis in the PdSe_2_ FET can be effectively suppressed. These strategies offer viable methods to reduce hysteresis in devices. Moreover, the types of charge carriers can be converted from *n*-type to *p*-type, which can be used as a switch for practical circuits.

Remarkably, vacuum annealing is an effective method for improving the mobility of FETs, and it has been widely used in electronic devices [72]. For PdSe_2_ FETs, annealing can remove the surface adsorbates to achieve higher mobility, on/off ratio, and lower Schottky barrier. Moreover, annealing at 450 K can eliminate hysteresis in FETs [17]. After annealing at 400 and 450 K, PdSe_2_ FET exhibits a higher mobility of 75 and 216 cm^2^ V^−1^ s^−1^ than that measured at room temperature. Such a transistor achieves the highest current ON/OFF ratio of 10^3^ at 450 K. Meanwhile, the threshold voltage shows an increasing shift to the negative gate voltage as the annealing temperature increases. This indicates that the Fermi level moved to the conduction band in PdSe_2_. Thus, the PdSe_2_ FET exhibits an obvious *n*-type transfer characteristic.

Furthermore, a molecular doping method converts the electron transport behavior of PdSe_2_ into a hole-transport feature [17]. As a prevalent *p*-dopant, F4-TCNQ has high electron affinity and has been utilized in low-dimensional materials [73], which can be applied to PdSe_2_. One can compare the transfer curves of the FET with different doping levels from undoped to completely doped, whereby a distinct conversion of the transfer characteristics from ambipolar to *p*-type is shown. The contact resistance of the PdSe_2_ FET has a dependence on the gate voltage, resulting from the Fermi level being adjusted by electrostatic gating. Therefore, vacuum annealing and molecular doping can effectively reduce contact resistance.

The transport properties of PdSe_2_ FETs can be altered via annealing or charge doping. Besides, the ambipolar behavior of PdSe_2_ FETs can be obtained by varying the electrical stress, pressure, and electron irradiation [70].

Atmospheric pressure has a significant influence on the PdSe_2_ FET. The transfer curves of the PdSe_2_ FET were extracted under various pressures at a constant drain voltage of 100 mV. As the pressure increases, the transfer nature of the PdSe_2_ FET gradually transforms from the *n*-type to the *p*-type, and the PdSe_2_ FET exposed to air after 10 min becomes a *p*-type depletion mode transistor [70].

Moreover, electron irradiation changes the charge distribution in the PdSe_2_ FET, which further affects the transfer characteristics [74]. The transfer curves of the PdSe_2_ FETs were collected before and after electron irradiation via SEM imaging. With increasing time after SEM imaging, the transfer characteristics slowly revert to the initial state.

Two-dimensional PdSe_2_ synthesized using different approaches has been used in the fabrication of FETs. Table 5 compares the performance of these PdSe_2_ FETs in terms of charge carrier mobility and current ON/OFF ratio. Further developments with large-area CVD-grown PdSe_2_ may improve the electrical performance, such as the charge mobility and ON/OFF ratio.

**Table 5:** The performances of field-effect transistors based on PdSe_2_ obtained from different methods

Synthesis methods and PdSe_2_ types	Electrode types	Charge mobility (cm^2^ V^−1^ s^−1^)	Current ON/OFF ratio	References
CVD (Domains)	Cr/Au	294	10^3^	[16]
Exfoliated (flake)	Ti/Au	158	10^6^	[15]
CVD (Domains)	Ti/Au	6.4	> 10^6^	[63]
Exfoliated (flake)	Ti/Au	4	10^4^	[19]
Exfoliated (flake)	Pd/Au	20	10^2^	[81]
Exfoliated (flake)	Pd_17_Se_15_/Ti/Au	170	n.a	[52]
Exfoliated (flake)	Ti/Au	8	n.a	[52]
Exfoliated (flake)	Ti/Au	216	10^3^	[17]
CVD (domains)	Cr/Au	n.a	10^3^	[140]
Exfoliated (flake)	Ti/Au	4	25	[70]
Exfoliated (flake)	Ti/Au	3	30	[74]
Exfoliated (flake)	Ti/Au	92	10^4^	[41]
Exfoliated (flake)	Ti/Au	138.9	10^3^	[54]

In summary, several strategies have been developed to improve the FET performance of PdSe_2_. Future opportunities still exist in terms of surface cleaning and modification, electrode contact design, packaging conditions, and vdWH stacking. Indeed, the PdSe_2_-based electronic devices could be integrated with the piezoelectric materials, i.e., PVDF for tactile sensors [75]. For the comfort of human beings, stretchable and wearable electronics become emerging with device development such as strain sensors and electronic skin [76]. Besides, the introduction of triboelectric nanogenerators, supercapacitors [77], and batteries [78] may lead to self-powered sensors [79].

After knowing the electronic devices of PdSe_2_, we turn to the progress in its applications in optoelectronics and optics.

## PdSe_2_ for Optoelectronics and Optics

The photodetector, which is a device that converts an optical signal into an electrical signal instantaneously, plays an indispensable role in current and burgeoning technology, in the fields of biotechnology, medicine, physics, and natural sciences [80].

Owing to their unique and significant properties, 2D materials have been applied in photodetectors and exhibit remarkable performance in terms of responsivity (R), detectivity (D*), and external quantum efficiency (EQE) [81]. Here, the responsivity *R* describes the photoelectric conversion efficiency, D* reflects the ability to measure the minimum optical signal, and EQE is the ratio of the number of photo-generated electron–hole pairs contributing to photocurrent to the number of the incident photons. The rise/fall time is a crucial parameter for evaluating the response speed of photodetectors.

Two-dimensional materials can be used as outstanding photodetector components by constructing heterojunctions [82] and gate-voltage regulated phototransistors [41]. For example, infrared photodetectors can employ the sensing materials such as BP [83], PtTe_2_ [10], and WS_2_. But h-BN, graphene/Si [84], and MoS_2_/GaN [85] can be used for ultraviolet light detection. Besides, PtSe_2_ has a large photoresponse at a wide spectral band ranging from 200 to 1550 nm [7]. Then, the anisotropic compounds such as PdSe_2_ can be used for polarized sensitive photoelectric detection [29]. Therefore, the coupling of PdSe_2_ and other 2D materials may cover the light detection of a broad spectral range.

In this section, we will discuss the detection band versus bandgap, photodetection performances, and polarized light detection based on PdSe_2_ and related materials.

### Detection Bands versus Bandgap

The performance of the photodetectors can be determined by the bandgaps of the materials. Photodetectors function at various wavelengths based on different 2D materials. Owing to the different bandgaps of the 2D materials, the corresponding photodetectors function in different spectral bands (Table 6).

**Table 6:** Detection bands of photodetectors and bandgaps depending on the types of 2D materials

Material	Bandgap (eV)	References	Detection bands		References
PdSe_2_	0–1.3	[141]	532	1060	[19]
	0–1.43	[15]	n.a	1060	[41]
PtS_2_	0.25–1.6	[142]	500	n.a	[143]
	0.25–1.6	[144]	405	1550	[144]
PtSe_2_	0.3–1.2	[106]	632	10,000	[106]
	0–1.17	[142]	n.a	980	[11]
ReS_2_	1.5	[145]	633	n.a	[145]
	1.5	[146]	405	655	[146]
ReSe_2_	1.27	[147]	520	n.a	[147]
	1.2–1.3	[148]	n.a	808	[148]
InSe	1.26	[149]	254	850	[149]
	1.26	[150]	365	685	[150]
In_2_Se_3_	1.3	[151]	500	800	[152]
	1.3	[153]	300	1100	[153]
AsP	0.15–0.3	[105]	2360	8050	[105]
	0.1–0.3	[154]	980	n.a	[154]
SnS	1.0–1.2	[155]	400	1050	[155]
SnS_2_	2.1	[156]	457	633	[156]
SnSe	1.30–1.55	[155]	400	1400	[155]
SnSe_2_	1–2	[86]	300	2000	[86]
Graphene	0	[157]	n.a	1550	[157]
	0	[158]	285	1150	[159]
	0	[160]	630	10,000	[160]
WS_2_	1.4–2.1	[161]	365	650	[161]
	1.4–2	[162]	650	690	[163]
WSe_2_	1.63	[164]	500	900	[164]
	1.2	[165]	473	1550	[166]
MoS_2_	1.35–1.82	[167]	375	808	[168]
	1.65	[169]	532	1070	[169]
MoSe_2_	8.4–1.1	[170]	638	n.a	[170]
	1.1	[171]	n.a	785	[172]
Phosphorene	0.3	[173]	532	3390	[174]
	0.3	[175]	400	3800	[176]

The performances of 2D material-based photodetectors can be determined as per details, such as black phosphorene or black phosphorus, MoS_2_, MoSe_2_, WS_2_, WSe_2_, graphene, SnS, SnSe, SnS_2_, SnSe_2_[86], InSe, In_2_Se_3_, ReS_2_, black AsP, PtSe_2_, PtS_2_, and PdSe_2_.

The PdSe_2_ layered material has remarkable optoelectronic properties, with a large bandgap tenability and extraordinary carrier mobility. The PdSe_2_-based devices are relatively stable and can be applied for photodetection from deep ultraviolet to mid-infrared bands [21], and the longest photodetection wavelength studied thus far is 10.6 μm [54].

### PdSe_2_ Photodetectors for Near-infrared Light Detection

The near-infrared light (1060 nm) is important for optical data communication and biomedical imaging. The small bandgap of monolayer PdSe_2_ features resonant optical absorption of such a wavelength. Therefore, PdSe_2_ is an ideal material for near-infrared light photodetectors.

A typical PdSe_2_ photodetector has been measured under monochromatic illumination [19]. Because the PdSe_2_ photodetector is based on field-effect transistors, the gate voltage plays an important role in photodetection. The responsivity of the PdSe_2_ photodetector demonstrates a strong gate voltage dependence under 1.06-μm light illumination. The device showed an ultrahigh responsivity of 708 A W^−1^ at a gate voltage of 30 V, and the detectivity was calculated to be 1.31 × 10^9^ Jones.

The normal positive trend of the photocurrent increases with increasing power intensity [19]. The responsivity of the PdSe_2_ photodetector under 4.05-μm illumination is much lower at 1.9 mA W^−1^. The photodetector exhibits excellent stability and repeatability in the environment at room temperature. The absorption spectra of PdSe_2_ flakes with different thicknesses demonstrate that the thick PdSe_2_ flakes have a higher MIR wavelength absorption. Therefore, this proves the feasibility of photodetection in the mid-infrared band.

However, the photoresponse time of PdSe_2_ photodetectors, in the order of several milliseconds, is less than desirable. The photogating effect may account for this phenomenon. That is, photogenerated electrons cannot recombine in a timely manner with photogenerated holes trapped by trap states. Therefore, the lifetime of photoelectrons is prolonged, and the device response is slow.

Both 2D materials and traditional 3D semiconductor materials can form heterostructures with PdSe_2_ and perform well in photodetection. A pyramid microstructure for heterojunction photodetectors have demonstrated their excellent performances via the light trapping effect and numerical modeling [62].

The PdSe_2_/pyramid Si photodetector can achieve greater performance than that of the PdSe_2_/Si photodetector in terms of the responsivity, detectivity, and ON/OFF ratio [62], and they are compared with other heterostructures (discussed later in 6.3). The PdSe_2_/pyramid Si photodetector can function as a self-driven device without a power supply. The tuning of the light intensity leads to a difference in the responsivity and ON/OFF ratio at zero bias. The maximum ON/OFF ratio can reach 1.6 × 10^5^. The responsivity and detectivity depend on the illuminating light wavelength, and the maximum values are 456 mA W^−1^ and 9.97 × 10^13^ Jones, respectively. Both are determined under 980-nm illumination for obtaining the peak sensitivity of the PdSe_2_/pyramid Si photodetector.

Similar to the Si pyramid, Ge nanocones (GeNCs) in heterojunction photodetectors can absorb photons more efficiently [87]. They have a higher photocurrent than that of the PdSe_2_/planar Ge heterostructure. Under 1550-nm illumination with a power intensity of 5 µW cm^−2^, the PdSe_2_/GeNCs photodetector exhibits a much larger responsivity (530.2 mA W^−1^) and quantum efficiency (42.4%) than those under 1300-nm and 1650-nm illumination. The variation of the current ON/OFF ratios with light intensity was compared under three different wavelengths. This proves the best performance of the PdSe_2_/GeNCs photodetectors in the 1550-nm detection.

### PdSe_2_ Photodetectors for Sensing Polarized Light

Polarized light detection can be achieved in the heterostructures of PdSe_2_ with other materials, such as PdSe_2_/Si nanowire arrays (SiNWA) [25] and PdSe_2_/perovskite [26] heterostructures.

**Fig. 19:**
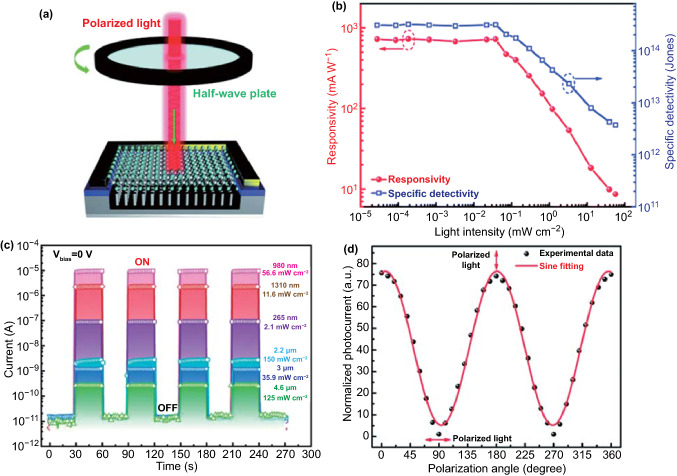
PdSe_2_ heterostructure-based photodetector for sensing polarized light. **a** Schematic illustration of photodetector based on PdSe_2_ and silicon nanowire arrays. **b** Light-intensity-dependent responsivity and detectivity of photodetector. **c** Time-dependent current of photodetector under illumination of infrared light with different wavelengths. Zero bias voltage applies. **d** Evolution of photocurrent under exposure of light with different polarization angles. The fitting curve approximates the sine function. Reprinted with permission from Ref. [25]. Copyright 2020, The Royal Society of Chemistry

Figure 19a demonstrates the schematic of the setup of the PdSe_2_/SiNWA heterostructure-based photodetector. The responsivity *R* and detectivity D* under various light intensities are shown in Fig. 19b. Both parameters increase with the decrease in light intensity and reach a maximum at 726 mA W^−1^ and 3.19 × 10^14^ Jones upon illumination with a light intensity of 27.5 cm^−2^. Notably, the PdSe_2_/SiNWA photodetector demonstrates a significant response to the weak light signals with a broad spectral detection range from the deep ultraviolet to the mid-infrared range (Fig. 19c).

However, it shows a high sensitivity to polarized light signals attributed to the asymmetric pentagonal structure of PdSe_2_. Here, the incident polarized light is supplied with various polarization angles through a half-wave plate using a polarizer. The normalized photocurrent was measured versus the polarization angle at zero bias (Fig. 19d). The polarization sensitivity of the PdSe_2_/SiNWA device is 75, which is higher than that of other 2D material-based devices.

Therefore, the PdSe_2_/SiNWA heterostructure exhibits great advantages as the self-driven and wide-band photodetector with highly polarization sensitivity. It has shown a remarkable broad photodetection from DUV to MIR with an excellent responsivity, specific detectivity, response time, and polarization sensitivity. Meanwhile, the device holds prominent potential in infrared imaging of high pixel resolution.

Under 650-nm illumination, the graphene/PdSe_2_/Ge photodetector [21] shows a record polarization sensitivity (112.2) among the reported PdSe_2_-based devices, including PdSe_2_/SiNWA photodetectors (75) and PdSe_2_/perovskite photodetectors (6.04) [26].

A comparison of polarized light sensing is presented for different 2D materials and their heterostructures (Table 7**)**. The polarization sensitivity of the graphene/PdSe_2_/Ge photodetector is much higher than that of some devices based on other 2D materials, such as GeS_2_ (2.1) [88], GeSe_2_ (2.16) [89], BP (8.7) [90], antimonene (17) [91], and BP/MoS_2_ heterostructures (22) [92].

**Table 7:** Polarization sensitivity performance of 2D material-based photodetectors

Material types	Illumination wavelength (nm)	Polarization sensitivity	References
Graphene/PdSe_2_/Ge	650	112.2	[21]
PdSe_2_/Si nanowire arrays	n.a	75	[25]
Phosphorene/MoS_2_	3500	22	[92]
Antimonene	450	17	[91]
Phosphorene	1550	8.7	[90]
PdSe_2_/perovskite	808	6.04	[26]
GeSe_2_	n.a	2.16	[89]
GeS_2_	325	2.1	[88]
GeSe	532	1.3	[177]
MoS_2_/GaAs	780	4.8	[178]
ZnSb	1342	1.28	[179]
PdSe_2_	532	1.29	[29]
PdS_2_	n.a	0	[180]
PtSe_2_	10,000	0	[106]
PtS_2_	500	0	[143]

Analogous to silicene and black phosphorus, PdSe_2_ has a high sensitivity to polarized light owing to its anisotropic crystalline structure. Based on this, graphene/PdSe_2_/Ge heterojunction photodetectors have been studied for the polarization-dependent photoresponse [21].

Overall, PdSe_2_-based photodetectors demonstrate remarkable photodetection of broadband bands (from deep ultraviolet to mid-infrared), good responsivity, outstanding stability, and sensitive polarization.

### PdSe_2_ Photodetector-enhanced Humidity Sensors

Besides image sensor, PdSe_2_-based devices can be applied to humidity sensors owing to the large surface-to-volume ratio of the PdSe_2_ film. For instance, a PdSe_2_/SiNWA device has been utilized as a highly sensitive sensor of the relative humidity (RH) of the ambient environment [25].

**Fig. 20:**
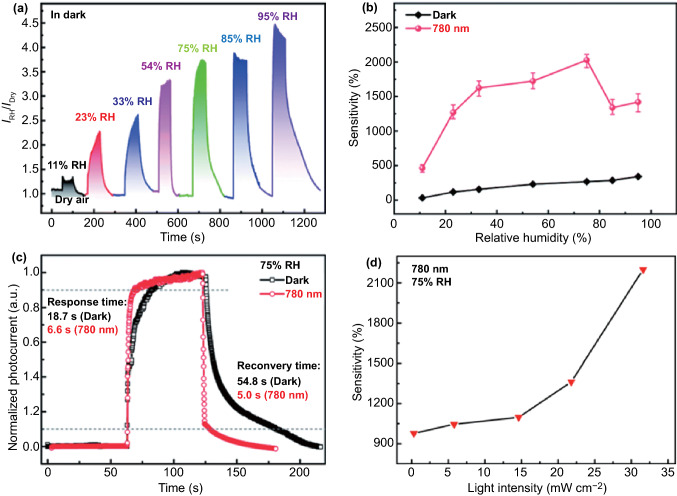
**a** Variation of current ratio of PdSe_2_-based device with relative humidity in the dark. **b** Relative humidity dependence of sensitivity in the dark and under 780-nm illumination. **c** Temporal response of PdSe_2_-based device at 75% RH in the dark and under 780-nm illumination. **d** Light intensity dependence of sensitivity at 75% RH under 780 nm. The RH denotes the relative humidity. Reprinted with permission from Ref. [25]. Copyright 2020, Royal Society of Chemistry

Figure 20a shows the response performance of the PdSe_2_/SiNWA devices at various relative humidity values from 11 to 95% in the dark. The response of the device exhibited good stability and repeatability at all RH values.

Moreover, the response of the PdSe_2_/SiNWA device under 780-nm illumination was significantly more sensitive than that in the dark (Fig. 20b). Figure 20c shows that the response speed is further improved under illumination when the RH value is 75%, and the response and recovery times are superior to those of some sensors based on other materials reported previously. The rapid response of the device under illumination may have resulted from the rapid recombination of carriers. Figure 20d plots the incident light intensity dependence of the sensitivity at 75% RH under 780-nm illumination, and the sensitivity of the device increases as the light intensity increases. Furthermore, the PdSe_2_/SiNWA device can retain its initial sensing performance after 6 months, indicating the good stability of the device [25].

### Saturable Absorber for Pulsed Laser

Graphene has been employed as a saturable absorber in the formation of pulsed lasers in the visible to mid-infrared range. However, the zero bandgap of graphene hinders its photonic application. Moreover, TMDCs have been employed as Q switches or mode lockers in the generation of pulsed lasers such as MoS_2_, WS_2_, MoSe_2_, and WSe_2_. However, their bandgaps are tunable in a limited range, i.e., from 1 to 2 eV, which suppresses the potential for application in optical regulation. With a wide range of tunable bandgaps, phosphorene has shown remarkable performance as a saturable absorber in pulsed lasers [93]. However, its weak air stability impedes further studies.

The tunable bandgap and air stability have guaranteed that PdSe_2_ is a saturable absorber (SA) in passive Q-switching, which is a crucial method when fabricating pulsed laser devices [94].

**Fig. 21:**
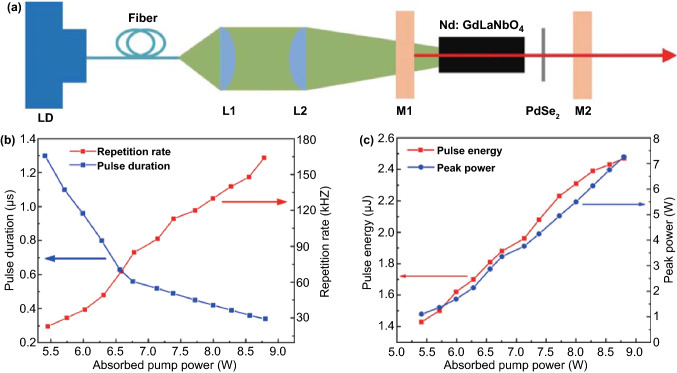
**a** Schematic illustration of PdSe_2_ passively Q-switched Nd:GdLaNbO_4_ pulsed laser experimental setup. **b** Pulse duration (left) and repetition rate (right) versus absorbed pump power. **c** Variation of the pulse energy (left) and pulse peak power (right) with the absorbed pump power. Reprinted with permission from Ref. [94]. Copyright 2020, Elsevier Ltd

A typical PdSe_2_-based passive Q-switched Nd:GdLaNbO_4_ laser is demonstrated (Fig. 21a). The laser diode (LD) as a direct pumping source is condensed into the Nd:GdLaNbO_4_ crystal through the fiber core and a pair of convex lenses (L1, L2), and it is then transformed into a pulsed laser through the PdSe_2_ nanosheet, while the plane mirrors (M1, M2) are coated with the transmission of different reflectivity to control the output laser.

The pulse repetition frequency shows a positive correlation with the absorbed pump power (Fig. 21b), whereas the pulse duration displays a negative correlation. Figure 21c shows the evolution of the pulse energy and peak power of the PdSe_2_/Nd:GdLaNbO_4_ laser with varying absorbed pump power, which may be due to the extensive modulation range of PdSe_2_. These results are better than those of MoS_2_ and WS_2_ [95], proving the excellent characteristics of the PdSe_2_ SA and the excellent potential of passive Q-switched lasers.

Due to the suitable bandgaps, 2D materials have been employed as saturable absorbers (SA) for passively Q-switched and mode-locked fiber laser. Besides, optical circuits have incorporated various saturable absorber materials, such as SnTe quantum dots, graphitic-phase C_3_N_4_, MoS_2_, PdS_2_, In_2_Se_3_, PtS_2_, WS_2_, and PdSe_2_ [96]. Indeed, they have emerged as cost-effective, simple, and highly integrated component for pulsed laser generation.

Future works may lie in the adoption of PdSe_2_-based van der Waals heterostructures as saturable absorbers for pulsed laser modulation in the fiber lasers or solid-state lasers.

Previously, the electronics, optoelectronics, and optics of PdSe_2_ have been introduced. Besides, the PdSe_2_ may possess great promises in the environmental, energy and biomedical applications. Indeed, the 2D materials have demonstrated the great performances in clean energy production [97–99], i.e., catalysis of hydrogen production or oxygen reduction, solar cells [100], thermoelectric power generation, energy storage, environmental remediation [101, 102], and photodegradation of organic-molecules-polluted water [103] as well as water purification. Besides, the metallic low dimension materials may favor the anti-bacterial performances as well as other biomedical engineering.

After knowing the devices of individual PdSe_2_ material, we come to the discussion of PdSe_2_-based van der Waals heterostructures.

## PdSe_2_-based van der Waals Heterostructures

The vdWHs of 2D materials employ weak layer interactions between two stacked layered materials to form multilayer structures. Owing to the enriched choice of conductivity types, 2D materials can be stacked by choosing from semiconducting, metallic, and insulating types. Indeed, 2D material-based vdWHs have enhanced the device architectures of conventional Si technology. Here, PdSe_2_ as a semiconducting 2D material could broaden the applicability of 2D vdWHs. In this section, we discuss emerging applications in electronics, such as rectifiers and optoelectronics, such as image sensors.

### Van der Waals Heterostructure Based on PdSe_2_/MoS_2_ Contact

**Fig. 22:**
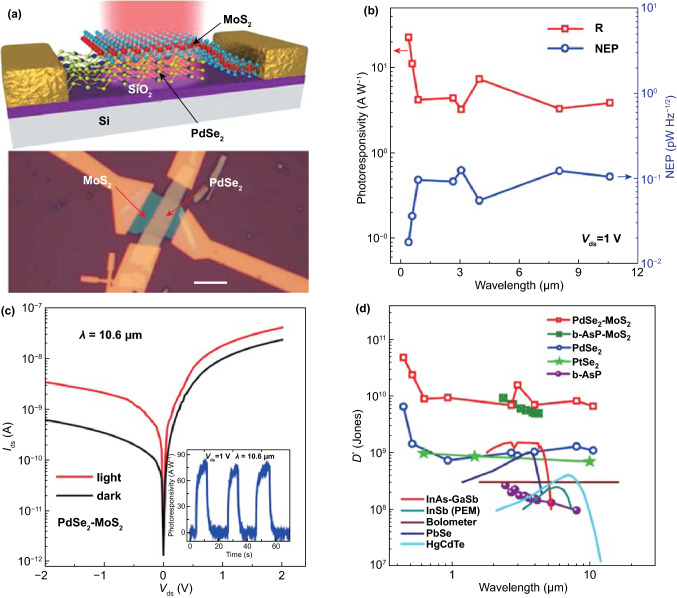
PdSe_2_/MoS_2_ van der Waals heterostructure-based photodetector. **a** Scheme of the PdSe_2_/MoS_2_ photodetector (top) and optical micrograph of the corresponding device (bottom), where the scale bar is 5 μm. **b** Wavelength dependence of photoresponsivity (red) and noise equivalent power (blue) of the PdSe_2_/MoS_2_ photodetector at V_DS_ = 1 V. NEP denotes noise equivalent power. **c** Drain current of heterostructure-based device under illumination and in the dark. The inset shows the current profile against time with periodic light illumination and dark state. **d** Wavelength-dependent detectivity of different 2D materials and some infrared materials at room temperature. Reprinted with permission from Ref. [54]. Copyright 2019, American Chemistry Society

Two-dimensional heterojunction-based photodetectors show superior photoresponse time and detectivity. PdSe_2_/MoS_2_ vdWH photodetectors (Fig. 22a) can effectively improve the responsivity and detectivity under 10.6-μm illumination, and the rise/fall time (τ_r_/τ_f_) of the photocurrent is 65.3/62.4 μs [54].

The vdWH can significantly suppress the dark current and current noise of the device, and the photocurrent can be generated under the combined action of intralayer excitons and interlayer excitons [54].

Figure 22b shows the responsivity and noise equivalent power as a function of the incident wavelength. PdSe_2_-based heterojunction photodetectors have better responsivity and detectivity for broadband detection. Typical photocurrent performances are presented in the dark and under illumination (Fig. 22c).

The photoresponse time can be improved by fast charge transfer in the heterostructure. Indeed, the PdSe_2_/perovskite heterojunction photodetector could solve the problems faced by most perovskite photodetectors, i.e., low specific detectivity and slow photoresponse [104].

The detectivity of PdSe_2_/MoS_2_ photodetectors can reach 8.21 × 10^9^ Jones, which is much better than that of most mid-infrared photodetectors (Fig. 22d) based on AsP [105], PtSe_2_ [106], graphene thermopiles [107], and uncooled HgCdTe [108]. The detectivity of PdSe_2_ exceeded that of some traditional mid-infrared photodetectors [108]. Compared with the PdSe_2_/MoS_2_ photodetector (Table 8), the potential of PdSe_2_ in mid-infrared photodetection is further reflected.

**Table 8:** Detectivity performance of 2D materials in mid-infrared photodetectors

Materials	Illumination wavelength (nm)	Detectivity (Jones)	References
PdSe_2_/MoS_2_	10,600	8.21 × 10^9^	[54]
AsP	5000	4.9 × 10^9^	[105]
PtSe_2_	10,000	7 × 10^8^	[106]
graphene thermopiles	n.a	8 × 10^8^	[107]
uncooled HgCdTe	9000	10^9^	[108]

### PdSe_2_ van der Waals Heterostructure-based ***p***–***n*** Junction-based Rectifier

The optoelectronics has stemmed from the fundamental component of *p*–*n* junctions. Indeed, the conventional 3D thin film stacking has contributed to the photovoltaics [109, 110], photodetectors, tunneling transistors, rectifiers, and light-emitting diodes. The metal/semiconductor contact has favored the Ohmic type conductance behavior for elevating the charge carrier transport. These investigations based on thin film deposition techniques have provided useful guide for 2D materials.

Two types of 2D materials stack together with weak interaction, termed van der Waals heterostructure. With delicate selection, one can assembly a *p*–*n* junction with the atomic layer thickness [111, 112]. No dangling bonds remain at their interface; besides, low lattice mismatch between both 2D materials result in the declined defect states. Therefore, the quantity of scattering center for charge carrier is minimized for boosting the charge carrier transport, which is superior to the Si based materials.

High gate-modulated rectification in vdWHs based on PdSe_2_ has been introduced and examined. For example, *p*-type germanium selenide (GeSe) and *n*-type PdSe_2_ with a pure ohmic contact show a large rectification ratio, which is defined as the ratio between the forward and reverse currents, up to 5.5 × 10^5^, resulting from the clean interface and low Schottky barrier [24].

**Fig. 23:**
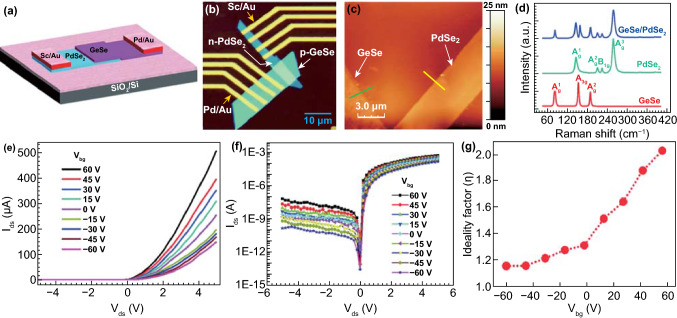
GeSe/PdSe_2_ junction-based rectifier. **a** Schematic of the GeSe/PdSe_2_ *p*–*n* junction. **b** Optical micrograph of the GeSe/PdSe_2_ junction with Pd/Au and Sc/Au electrodes. **c** AFM image of the GeSe/PdSe_2_ heterostructure. **d** Raman spectra of the GeSe/PdSe_2_ junction and individual flakes. **e, f** Drain current versus voltage curves of the device in a linear scale and semi-log scale with sweeping different back gate voltages. **g** Gate voltage dependence of the ideality factor of the device. Reprinted with permission from Ref. [24]. Copyright 2020, The Royal Society of Chemistry

One can find schematic of the *p*-GeSe/*n*-PdSe_2_ vdWH-based rectifier device (Fig. 23a), and the corresponding optical image (Fig. 23b).

Figure 23c displays the AFM images of the GeSe and PdSe_2_ flakes with thicknesses of 12 and 11.5 nm, respectively. The Raman spectra of GeSe and PdSe_2_ demonstrate the successful stacking of both 2D materials (Fig. 23d).

For *p*-GeSe/*n*-PdSe_2_ diodes, the linear scale (Fig. 23e) and the semi-log scale (Fig. 23f) of the drain current versus voltage curves were measured at different gate voltages. Indeed, the gate voltage can modulate the rectifying effect. This result is due to the carrier density and electrostatic inversion from semiconductor to semi-insulator materials [113]. Figure 23g presents the variation in the ideality factor η of the *p*-*n* diodes, which is obtained as 1.2 at a negative gate voltage. The *p*-*n* diode tends to decrease its ideality at a positive gate voltage, which can be attributed to the carrier recombination at the sharp interface resulting from the decrease in electric field [114]. Table 9 compares the rectification ratios of typical *p*-*n* diodes based on the vdWHs of PdSe_2_ and other 2D materials.

**Table 9:** Rectification ratio of *p*–*n* junction-based diodes with different van der Waals heterostructures based on 2D materials

*p*–*n* junction diodes	Rectification ratio	References
GeSe/PdSe_2_	5.5 × 10^5^	[24]
Phosphorene/PdSe_2_	7.1 × 10^5^	[117]
Phosphorene/MoS_2_	1 × 10^5^	[181]
GaSe/InSe	1 × 10^5^	[182]
Graphene/WSe_2_	1 × 10^4^	[115]
WSe_2_/MoS_2_	1 × 10^4^	[183]
WSe_2_/SnSe_2_	2.1 × 10^4^	[184]
MoS_2_/WSe_2_	1.3 × 10^5^	[185]
WSe_2_/GeSe	1 × 10^5^	[186]

This proves that nTMDC-based rectifier may hold promises in logic switches as shown in other TMDC logic circuits [115]. Besides, the nTMDC-based rectifier could be employed as an energy harvester for collecting the electromagnetic wave energy as proved by other 2D materials [116].

### PdSe_2_ van der Waals Heterostructure-based Junction Photodetectors

The PdSe_2_ based van der Waals heterostructures remain less investigated in terms of fabrication strategies. One can refer to the investigation of other vdWH emerging 2D materials. To date, the dry stamp transfer method has dominated the stacking nanosheets. Indeed, the epitaxy-based synthesis has great opportunities of fabricating the secondary layer of 2D materials. Besides, large quantity of 2D materials remain unexplored for the stacking of 2D materials such as metal–organic framework, graphene, MoS_2_, ReSe_2_, PtSe_2_, MXene, and tellurium as well as perovskites. Besides, the lateral heterostructure may arise the attention for novel charge carrier transport.

**Fig. 24:**
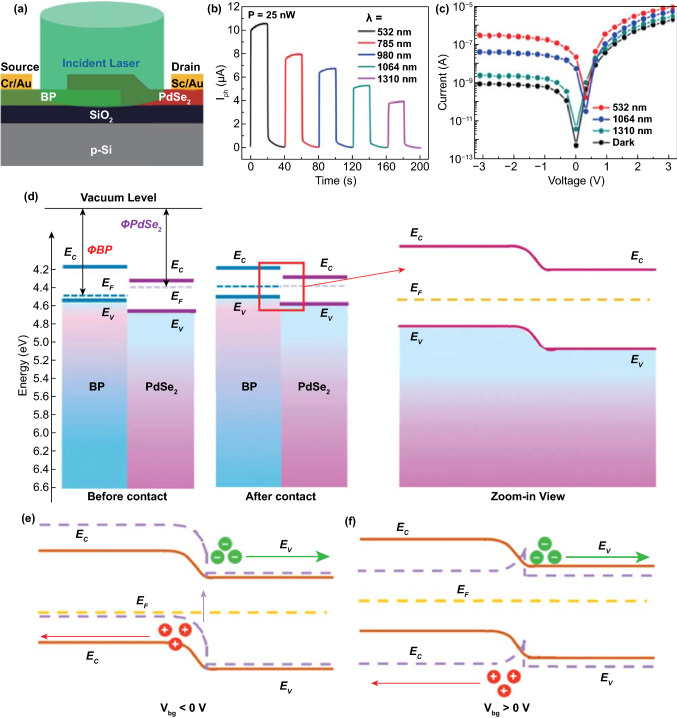
**a** Schematic of the *p*-BP/*n*-PdSe_2_ *p* − *n* diode under illumination. **b** Time-dependent I_ph_ of the *p*-BP/*n*-PdSe_2_ photodiodes under the illumination of different wavelengths. **c** I_DS_ − V_DS_ curves under the illumination of different wavelengths at back gate voltage of 10 V. **d** Energy band of the *p*-BP/*n*-PdSe_2_ van der Waals heterojunction before contact and after contact with a zoom-in view. Energy band alignment of a *p*-BP/*n*-PdSe_2_ photodiode under **e** a negative back-gate voltage and **f** a positive back-gate voltage. Reprinted with permission from Ref. [117]. Copyright 2020, American Chemical Society

To investigate the additional features of the *p*-*n* vdWH diode, the photoresponse was investigated [117]. Figure 24a shows a schematic of the *p*-BP/*n*-PdSe_2_ vdWH diode under illumination.

The time-resolved photocurrent was measured under intermittent lasers with different wavelengths at a fixed power (Fig. 24b). The varying incident wavelengths from the visible to NIR region on the *p*-BP/*n*-PdSe_2_ diode led to current versus voltage curves (Fig. 24c). This indicates that the photocurrent decreased when the incident wavelength increased. The photocurrent of the diode depends on the back-gate voltage (Fig. 24d).

The energy band alignment of *p*-BP, *n*-PdSe_2_, and their heterostructures after contact (Fig. 24e), with the CBM, VBM, work function, and electron affinity. A magnified view of the band alignment is presented after contact at a gate voltage of 0 V (Fig. 24e). When the diode operates at a negative back-gate voltage, the Fermi level moves away from the conductance band. This increases the potential barrier of the *p*-BP/*n*-PdSe_2_ interface, resulting in a high rectifying current. The Fermi level approaches the conductance band at a negative gate voltage (Fig. 24f) and decreases the potential barrier and rectification ratio. For the *p*-BP/*n*-PdSe_2_ diode, the positive gate voltage (Fig. 24g) can modulate the Fermi level and control the carrier densities, which can eventually control the rectification ratio.

Therefore, the PdSe_2_ *p*–*n* junction-based photodiode shows a great potential in high-performance visible-infrared photodetectors, as well as solar cell for electricity production. This *p*-*n* diode concept may broaden the application of 2D nTMDC-based heterostructures in photovoltaics.

In this section, we discuss the structure and performance of different photodetectors based on PdSe_2_. A comparison of the performances of different PdSe_2_-based photodetectors is listed in Table 10.

**Table 10:** Performances of photodetectors based on 2D PdSe_2_ and its van der Waals heterostructures as well as related nanostructures

Materials	Wavelength or band *λ* (nm)	Responsivity *R* (mA W^−1^)	Detectivity D* (Jones)	I_light_/I_dark_	τ_r_/τ_f_	References
PdSe_2_/MoS_2_	10,600	4.21 × 10^4^	8.2 × 10^9^	10	65.3/62.4 μs	[54]
PdSe_2_/GeNCs	1550	530.2	1.45 × 10^11^	7 × 10^3^	25.4/38.5 μs	[87]
PdSe_2_	1060	7.08 × 10^5^	1.31 × 10^9^	10	220 ms	[19]
PdSe_2_	1060	n.a	n.a	n.a	156/163 μs	[41]
PdSe_2_/Ge	980	691.5	1.73 × 10^13^	10^5^	6.4/92.5 μs	[21]
PdSe_2_/pyramid Si	980	456	9.97 × 10^13^	1.6 × 10^5^	n.a	[119]
PdSe_2_/SiNWA	980	726	3.19 × 10^14^	10^6^	3.4/3.9 μs	[25]
PdSe_2_/perovskite	808	313	2.72 × 10^13^	10^4^	3.5/4 μs	[26]
PdSe_2_/Si	780	300.2	1.18 × 10^13^	1.08 × 10^5^	38/44 μs	[62]
PtSe_2_/SiNWA	200–1550	1.265 × 10^4^	2.5 × 10^13^	4 × 10^4^	10.1/19.5 μs	[7]
PtSe_2_/*n*-GaN	265	193	3.8 × 10^14^	10^8^	45/102 μs	[82]
PtS_2_	500	1.56 × 10^6^	2.9 × 10^11^	n.a	0.46 s	[143]
WS_2_/*p*-Si	340–1100	5.7 × 10^3^	n.a	10.65	670/998 μs	[187]
WS_2_	365	5.35 × 10^4^	1.22 × 10^11^	n.a	n.a	[161]
NiPS_3_	254	126	1.22 × 10^12^	200	3.2/15.6 ms	[188]
BP	640–940	4.8	n.a	n.a	1/4 ms	[83]
MoS_2_/BP	532–1550	153	n.a	n.a	15/70 μs	[189]
MoS_2_/*n*-Si	300–1100	1.19 × 10^4^	2.1 × 10^10^	59.9	30.5/71.6 μs	[190]
MoS_2_/Graphene	420–980	835	n.a	n.a	20/30 ms	[191]
MoS_2_/*p*-GaN	265	187	2.34 × 10^13^	10^5^	46.4/114.1 μs	[85]
GaN	325	340	1.24 × 10^9^	n.a	280/450 ms	[192]
GaSe/GaSb	400–1800	115	1.3 × 10^12^	n.a	32/24 μs	[193]
InGaAs/*p*-Si	400–1250	7.52 × 10^3^	n.a	n.a	13/16 ms	[194]
MgO	150	1.86 × 10^3^	1.8 × 10^10^	10^2^	n.a	[195]
Graphene/Ge	1200–1600	51.8	1.38 × 10^10^	2 × 10^4^	23/108 μs	[196]
Graphene/*n*-Si	300–1100	730	4.2 × 10^12^	10^4^	320/750 μs	[197]
Graphene/MoTe_2_/Graphene	1064	110	10^10^	n.a	24/46 μs	[198]
Graphene/Si	365	120	6.1 × 10^12^	10^5^	4/12 ns	[84]

### Image Sensor System from PdSe_2_ van der Waals Heterostructure

Because of the excellent performance of PdSe_2_ in the field of photodetection, some studies subsequently explored further possibilities in the image sensor field. Infrared image sensors have emerged as an essential device unit in optoelectronic systems such as fire monitoring, night vision, and surveillance cameras [118].

The PdSe_2_/pyramid Si device presented superior results in terms of infrared image sensing [119]. In portable systems, cardboard masks can be imaged using such a device. The geometry of the house and tree shapes was imaged under 980-nm and 1300-nm illumination. The illuminated areas are highlighted in photocurrent mapping. In contrast, the photocurrent in the other areas remained much weaker, similar to the dark state.

Although some blemishes in the blocked regions need to be further corrected, the shapes of the patterns can be distinguished easily by contrast. Similar results were obtained for the PdSe_2_/GeNCs hybrid device [263], indicating the reliable infrared imaging capability of PdSe_2_-based devices.

The suitable bandgap of PdSe_2_ guarantees its application in infrared light sensing. When the devices are fabricated into arrays, the system can achieve image sensing with high pixel numbers [21]. When the polarized light is incident on the device through a specific mask, the lock-in amplifier can timely scan the voltage of the device and transform it into the voltage mapping image.

**Fig. 25:**
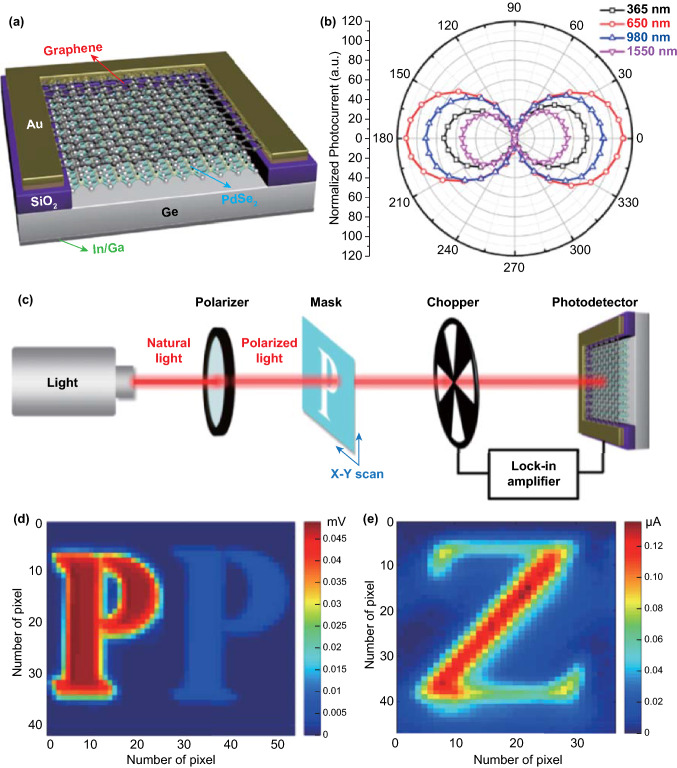
Graphene/PdSe_2_/Ge heterostructure-based polarized-light image sensor. **a** View of the image sensing device. **b** Photocurrent of the sensor under the illumination of monochromatic light by regulating different polarization angles. The light sources of four wavelengths are employed for light irradiation. **c** System setup for imaging the mask of a capital *P* with the illumination of infrared polarized light. **d** High-resolution current mapping image of the capital *P* under 780-nm light illumination with polarization angles of 0^°^ (left) and 90^°^ (right). **e** Imaging of a capital Z with a polarization angle of 0^°^. Reprinted with permission from Ref. [21] Copyright 2019, American Chemistry Society

The graphene/PdSe_2_/Ge photodetector with a broadband range from ultraviolet to near-infrared light (Fig. 25a).

The normalized photocurrent of the graphene/PdSe_2_/Ge device shows a strong correlation with the polarization angle under various illuminations having different wavelengths, including 365, 650, 980, and 1550 nm (Fig. 25b).

The maximum and minimum values of the photocurrent were achieved at polarization angles of 0° (180°) and 90° (270°), respectively. Indeed, the sine-shaped photocurrent curve indicates a good polarization sensitivity of the graphene/PdSe_2_/Ge device [21].

A high-resolution infrared image is compiled after projecting a patterned light to the detector (Fig. 25c) after passing through the *P* letter mask (Fig. 25d). Here, the photocurrent intensity is stronger with a polarization angle of 0° (left panel) than that of polarized light at 90° (right panel). The high polarization contrast ratio (> 10) between the polarization angles of 0° and 90° indicates the outstanding performance of the PdSe_2_-based device in polarized light imaging. The heterojunction-based photodetector has excellent potential as a mid-infrared image sensor. Figure 25e presents a highly recognizable spectral image of the Z letter under 3043-nm illumination with a polarization angle of 0^°^.

Such an image sensor is highly promising for broadband photodetection and imaging. The PdSe_2_ heterojunction-based photodetector demonstrates an extraordinary polarization sensitivity, which is the highest value among 2D material-based polarized light photodetectors (thus far). On account of a strong asymmetry of PdSe_2_, the effective separation of photogenerated electron–hole pairs occurs by a built-in perpendicular electric field in the *p*–*n* junction. Then, the efficiency of the carrier collection is enhanced by graphene electrode. Therefore, PdSe_2_ is a very profound material for high-performance polarization-sensitive photodetectors.

**Fig. 26:**
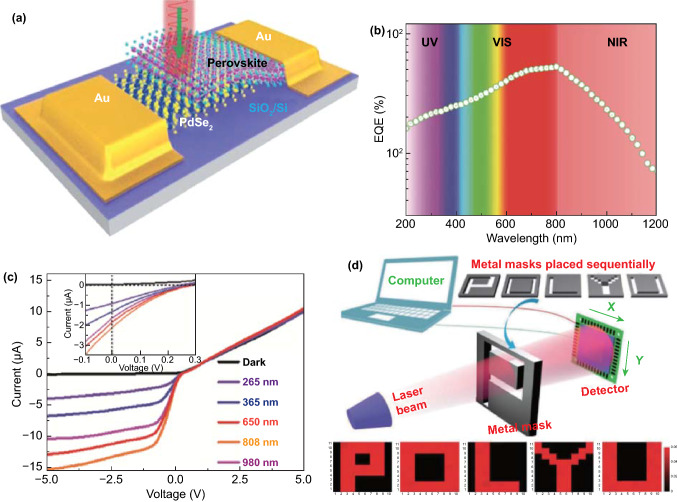
PdSe_2_/perovskite heterostructure-based photodetector arrays for imaging. **a** Schematic of the PdSe_2_/perovskite-based photodetector. **b** External quantum efficiency of the PdSe_2_/perovskite device as a function of the incident wavelength. **c** Current–voltage curve of the PdSe_2_/perovskite photodetector in the dark and under illumination with different wavelengths. Inset is zoomed-in current–voltage curves at the range from –0.1 to 0.3 V. **d** Scheme of the imaging system based on the photodetector arrays under 808-nm illumination, and 2D photocurrent mapping images after sensing five different letters. Adapted under the terms of the Creative Commons CC by license (https://creativecommons.org/licenses/by/4.0/) from Ref. [26] Copyright 2019, The Authors, published by WILEY–VCH Verlag GmbH & Co. KGaA, Weinheim

The integration with light-absorbing materials could provide the power source owing to the photovoltaic effect. Moreover, the use of perovskite as an absorber material can transform the light into electricity for self-powering by forming a Schottky junction with PdSe_2_. The PdSe_2_/perovskite heterostructure photodetector is illustrated in Fig. 26a with a high quantum efficiency (Fig. 26b).

Such a photodetector has a broad detection band ranging from 200 to 1200 nm. Through tuning of the illuminating light with various incident wavelengths from 265 to 980 nm, the current of the photodetector has been recorded. Here, the photocurrent curve displays a maximum value under 808-nm illumination at a negative bias (Fig. 26c), which corresponds to the quantum efficiency peak around 800 nm.

In the inset panel, the current–voltage curves show the photovoltaic effect upon illumination. Similarly, PdSe_2_/perovskite photodetector-based arrays can be employed in image sensing with significant photoresponse capability (Fig. 26d). When the infrared light passes through the mask, the projection of the features from the mask is captured by the detector. Moreover, the processing unit converted the current signal to each pixel. Subsequently, the 2D contrast current mapping software automatically incorporates the data and exhibits the current mapping image.

Consequently, the outlines of the five letters can be recognized by 2D current mapping under 808-nm illumination. Therefore, the PdSe_2_/perovskite heterostructure device shows potential for the future image sensing of complicated shapes such as human beings and animals.

The currently available image sensors are listed in Table 11. The resolution and current contrast ratio may require the future efforts for improvement.

**Table 11:** Performances of image sensors based on 2D PdSe_2_ and its van der Waals heterostructures

Material types	Resolution (pixel)	Mask area (cm^2^)	Active device area (cm^2^)	Current contrast ratio	Wavelength *λ* (nm)	References
PdSe_2_/Ge	46 × 46	5 × 5	0.6 × 0.6	> 10^2^	3043	[21]
PdSe_2_/pyramid Si	19 × 20	several	0.1 × 0.1	> 10^2^	1330	[119]
PdSe_2_/GeNCs	6 × 6	several	Sub-1	10^2^	1550	[87]

Owing to the superior capability of detecting mid-infrared light at room temperature, PdSe_2_-based devices highlight the high potential for application of photodetectors and image sensor systems. There remain good opportunities in the formation and application of vdWHs based on PdSe_2_ and other 2D materials. Indeed, low-dimensional materials have yet to be tested in vdWH assemblies with the coupling of PdSe_2_.

## Conclusions and Outlook

In this work, we deliver a comprehensive review of the progress in the rising-star pentagonal 2D material, i.e., palladium diselenide. First, the fundamental of PdSe_2_ is introduced with the types, atomic and electronic structure, bandgap, and vibration properties. Second, the synthesis approaches are listed with top-down and bottom-up methods. Indeed, the authors are fed with mechanical exfoliation, plasma thinning, and vacuum annealing. Then, the large-area synthesis has been introduced with thermal selenization of Pd thin film, and chemical vapor deposition with different Pd precursors such as PdCl_2_ powers. Third, the electronic and optoelectronic devices are discussed with the metal/semiconductor contact, field-effect transistors, photodetectors, and humidity sensors. The PdSe_2_ has been employed in the generation of pulsed laser and the thermoelectric power. Last but not the least, the van der Waals heterostructures of PdSe_2_ are delivered as well as their applications in the rectifier, photodetectors, and image system.

The fundamental physics of PdSe_2_ may provide for the insight for the guide of device design and fabrication. Indeed, the engineering applications of conventional devices and characterization tools require refreshing novel materials to enrich the interdisciplinary research across the microelectronics, optoelectronics, spectroscopy, optics, photonics, spintronics, and valleytronics. Besides, the magnetic properties of materials are interesting for the incubation of the proof-of-concept devices. Besides, the band alignment in a heterostructure may provide a platform for photo-generated carrier transport. The 2D materials as saturable absorbers have demonstrated extraordinary performances in Q-switching and mode lock for pulsed laser generation. Indeed, the metallic 2D materials have demonstrated superior performances in electromagnetic interference shielding or microwave absorption. Besides, the incorporation of magnetic nanoparticles may lead to the change of magnetoresistance as a magnetic field sensor.

The performance of PdSe_2_ devices has been verified in photodetectors[62], field-effect transistors [16], and humidity sensors [25]. First, PdSe_2_ transistors demonstrate pressure-tunable hysteresis [70], field emission [81], and phototransistors [41]. Second, the narrow bandgap of PdSe_2_ guarantees its performance in an infrared range such as 10.6-µm light detection [54] and broadband sensing [19]. Third, the linear dichroism transition [39] in PdSe_2_ guarantees optical switching and communication. As a saturable absorber, PdSe_2_ shows success in Q-switching for pulsed lasers [96].

There are still plenty of room in the development of sophisticated techniques for mass production of PdSe_2_. First, the chemical vapor deposition [120] has the features of upscale production, large-area homogeneity, and compatibility with Si-based technology. Indeed, the chemical vapor deposition of 2D materials [121–123] become necessary to achieve the synthesis over a large area and even a wafer size [124]. Owing to the layer-dependent properties, the preparation methods of high-quality 2D PdSe_2_ should be modified to accurately control the thickness, which is essential for the manufacture of high-performance devices. According to the trend of sophisticated 2D materials such as graphene, the quality of synthetic PdSe_2_ may go through the path, i.e., from mechanically exfoliated nanosheets, ball-milled nano-powders [125], polycrystalline thick films, monolayer or bilayer polycrystalline thin film [126, 127], and monolayer single-crystal domains [128]. More effective synthesis of atomically thin, large-scale, and uniform 2D PdSe_2_ should be explored to satisfy the needs of industrialization. Eventually, the domain size of PdSe_2_ single crystal may expand to centimeter scale and even to the wafer scale.

The posttreatment of PdSe_2_ may broaden its material properties, and consequently, its device performances may vary. First, the thermal annealing or plasma treatment [129] has shown modification of 2D materials. Second, the in situ characterization tools such as transmission electron microscopy [130] and XRD may provide the direct evidence for phase transition, i.e., the lattice distortion in the atomic scale. Third, the machine learning acts an efficient tool for defects determination and device performances enhancement. The properties of 2D materials could be regulated with defect engineering by the theoretical calculation, as well as big data for materials science. Besides, the patterning of 2D materials becomes a prerequisite for the fabrication of device arrays.

Future opportunities of PdSe_2_-based devices and systems remain great at integrated circuits as well as the internet of things. Indeed, the 2D materials have been incorporated in the logic gate-based digital circuits, programmable memories, and RF integrated circuits. One can refer to the graphene [124] and transition-metal dichalcogenides [131] for borrowing the concept of heterostructures. There remains a vortex of materials science research for artificial intelligence such as actuator devices, and human/machine interface. Therefore, great prospects of PdSe_2_-based van der Waals heterostructures are calling for the input of physicists, chemists, and materials scientists as well as industrial engineers.

## References

[1] Saito Y, Ge J, Watanabe K, Taniguchi T, Young AF (2020). Independent superconductors and correlated insulators in twisted bilayer graphene. Nat. Phys..

[2] Jin C, Kim J, Utama MIB, Regan EC, Kleemann H (2018). Imaging of pure spin-valley diffusion current in WS_2_-WSe_2_ heterostructures. Science.

[3] Pang Y, Yang Z, Yang Y, Ren TL (2020). Wearable electronics based on 2D materials for human physiological information detection. Small.

[4] Agrawal AV, Kumar N, Kumar M (2021). Strategy and future prospects to develop room-temperature-recoverable NO_2_ gas sensor based on two-dimensional molybdenum disulfide. Nano-Micro Lett..

[5] Holden NE, Coplen TB, Böhlke JK, Tarbox LV, Benefield J (2018). IUPAC periodic table of the elements and isotopes (IPTEI) for the education community (IUPAC Technical Report). Pure Appl. Chem..

[6] Mak KF, Shan J (2016). Photonics and optoelectronics of 2D semiconductor transition metal dichalcogenides. Nat. Photon..

[7] Zeng L, Lin S, Lou Z, Yuan H, Long H (2018). Ultrafast and sensitive photodetector based on a PtSe_2_/silicon nanowire array heterojunction with a multiband spectral response from 200 to 1550 nm. NPG Asia Mater..

[8] Kempt R, Kuc A, Heine T (2020). Two-dimensional noble-metal chalcogenides and phosphochalcogenides. Angew. Chem. Int. Ed..

[9] Ahmad S (2017). Strain dependent tuning electronic properties of noble metal di chalcogenides PdX_2_ (X = S, Se) mono-layer. Mater. Chem. Phys..

[10] Zeng L, Wu D, Jie J, Ren X, Hu X (2020). Van der Waals epitaxial growth of mosaic-like 2D platinum ditelluride layers for room-temperature mid-infrared photodetection up to 10.6 microm. Adv. Mater..

[11] Zhao Y, Qiao J, Yu Z, Yu P, Xu K (2017). High-electron-mobility and air-stable 2D layered PtSe_2_ FETs. Adv. Mater..

[12] Yang H, Li Y, Yang Z, Shi X, Lin Z (2020). First-principles calculations of the electronic properties of two-dimensional pentagonal structure XS_2_ (X=Ni, Pd, Pt). Vacuum.

[13] Saraf D, Chakraborty S, Kshirsagar A, Ahuja R (2018). In pursuit of bifunctional catalytic activity in PdS2 pseudo-monolayer through reaction coordinate mapping. Nano Energy.

[14] Ghorbani-Asl M, Kuc A, Miro P, Heine T (2016). A single-material logical junction based on 2D Crystal PdS_2_. Adv. Mater..

[15] Oyedele AD, Yang S, Liang L, Puretzky AA, Wang K (2017). PdSe_2_: pentagonal two-dimensional layers with high air stability for electronics. J. Am. Chem. Soc..

[16] Gu Y, Cai H, Dong J, Yu Y, Hoffman AN (2020). Two-dimensional palladium diselenide with strong in-plane optical anisotropy and high mobility grown by chemical vapor deposition. Adv. Mater..

[17] Chow WL, Yu P, Liu F, Hong J, Wang X (2017). High mobility 2D palladium diselenide field-effect transistors with tunable ambipolar characteristics. Adv. Mater..

[18] Puretzky AA, Oyedele AD, Xiao K, Haglund AV, Sumpter BG (2018). Anomalous interlayer vibrations in strongly coupled layered PdSe_2_. 2D Mater..

[19] Liang Q, Wang Q, Zhang Q, Wei J, Lim SX (2019). High-performance, room temperature, ultra-broadband photodetectors based on air-stable PdSe_2_. Adv. Mater..

[20] Yang H, Kim SW, Chhowalla M, Lee YH (2017). Structural and quantum-state phase transitions in van der Waals layered materials. Nat. Phys..

[21] Wu D, Guo J, Du J, Xia C, Zeng L (2019). Highly polarization-sensitive, broadband, self-powered photodetector based on graphene/PdSe_2_/germanium heterojunction. ACS Nano.

[22] Tai KL, Chen J, Wen Y, Park H, Zhang Q (2020). Phase variations and layer epitaxy of 2D PdSe_2_ GRown on 2D monolayers by direct selenization of molecular Pd precursors. ACS Nano.

[23] Jakhar M, Singh J, Kumar A, Tankeshwar K (2020). Pressure and electric field tuning of Schottky contacts in PdSe_2_/ZT-MoSe_2_ van der Waals heterostructure. Nanotechnology.

[24] Afzal AM, Iqbal MZ, Mumtaz S, Akhtar I (2020). Multifunctional and high-performance GeSe/PdSe2 heterostructure device with a fast photoresponse. J. Mater. Chem. C.

[25] Wu D, Jia C, Shi F, Zeng L, Lin P (2020). Mixed-dimensional PdSe_2_/SiNWA heterostructure based photovoltaic detectors for self-driven, broadband photodetection, infrared imaging and humidity sensing. J. Mater. Chem. A.

[26] Zeng LH, Chen QM, Zhang ZX, Wu D, Yuan H (2019). Multilayered PdSe_2_/perovskite schottky junction for fast, self-powered, polarization-sensitive, broadband photodetectors, and image sensor application. Adv. Sci..

[27] Sun J, Shi H, Siegrist T, Singh DJ (2015). Electronic, transport, and optical properties of bulk and mono-layer PdSe_2_. Appl. Phys. Lett..

[28] Grønvold F, Røst E (1957). The crystal structure of PdSe_2_ and PdS_2_. Acta Crystallogr..

[29] Zhong J, Yu J, Cao L, Zeng C, Ding J (2020). High-performance polarization-sensitive photodetector based on a few-layered PdSe_2_ nanosheet. Nano Res..

[30] Zhao Y, Qiao J, Yu P, Hu Z, Lin Z (2016). Extraordinarily strong interlayer interaction in 2D layered PtS_2_. Adv. Mater..

[31] Kuklin AV, Ågren H (2019). Quasiparticle electronic structure and optical spectra of single-layer and bilayer PdSe_2_: Proximity and defect-induced band gap renormalization. Phys. Rev. B.

[32] Zhao X, Zhao Q, Zhao B, Dai X, Wei S (2020). Electronic and optical properties of PdSe_2_ from monolayer to trilayer. Superlattices Microstr..

[33] Lei W, Cai B, Zhou H, Heymann G, Tang X (2019). Ferroelastic lattice rotation and band-gap engineering in quasi 2D layered-structure PdSe_2_ under uniaxial stress. Nanoscale.

[34] Zhao X, Qiu B, Hu G, Yue W, Ren J (2018). Spin polarization properties of pentagonal PdSe(2) induced by 3D transition-metal doping: first-principles calculations. Materials.

[35] Zhang S-H, Liu B-G (2018). Hole-doping-induced half-metallic ferromagnetism in a highly-air-stable PdSe2 monolayer under uniaxial stress. J. Mater. Chem. C.

[36] Deng S, Li L, Zhang Y (2018). Strain modulated electronic, mechanical, and optical properties of the monolayer PdS_2_, PdSe_2_, and PtSe_2_ for tunable devices. ACS Appl. Nano Mater..

[37] Liu G, Zeng QM, Zhu PF, Quhe RG, Lu PF (2019). Negative Poisson's ratio in monolayer PdSe_2_. Comput. Mater. Sci..

[38] ElGhazali MA, Naumov PG, Mirhosseini H, Suss V, Muchler L (2017). Pressure-induced superconductivity up to 13.1 K in the pyrite phase of palladium diselenide PdSe2. Phys. Rev. B.

[39] Yu J, Kuang X, Gao Y, Wang Y, Chen K (2020). Direct observation of the linear dichroism transition in two-dimensional palladium diselenide. Nano Lett..

[40] Lei W, Zhang S, Heymann G, Tang X, Wen J (2019). A new 2D high-pressure phase of PdSe_2_ with high-mobility transport anisotropy for photovoltaic applications. J. Mater. Chem. C.

[41] Walmsley TS, Andrews K, Wang T, Haglund A, Rijal U (2019). Near-infrared optical transitions in PdSe_2_ phototransistors. Nanoscale.

[42] Sun M, Chou JP, Shi L, Gao J, Hu A (2018). Few-Layer PdSe_2_ sheets: promising thermoelectric materials driven by high valley convergence. ACS Omega.

[43] Cai Y, Zhang G, Zhang YW (2014). Polarity-reversed robust carrier mobility in monolayer MoS(2) nanoribbons. J. Am. Chem. Soc..

[44] Ge X-J, Qin D, Yao K-L, Lü J-T (2017). First-principles study of thermoelectric transport properties of monolayer gallium chalcogenides. J. Phys. D-Appl. Phys..

[45] Nguyen GD, Liang L, Zou Q, Fu M, Oyedele AD (2018). 3D imaging and manipulation of subsurface selenium vacancies in PdSe_2_. Phys. Rev. Lett..

[46] Lin J, Zuluaga S, Yu P, Liu Z, Pantelides ST (2017). Novel Pd_2_Se_3_ two-dimensional phase driven by interlayer fusion in layered PdSe_2_. Phys. Rev. Lett..

[47] Chen J, Ryu GH, Sinha S, Warner JH (2019). Atomic structure and dynamics of defects and grain boundaries in 2D Pd_2_Se_3_ Monolayers. ACS Nano.

[48] Zuluaga S, Lin J, Suenaga K, Pantelides ST (2018). Two-dimensional PdSe_2_-Pd_2_Se_3_ junctions can serve as nanowires. 2D Mater..

[49] Ryu GH, Zhu T, Chen J, Sinha S, Shautsova V (2019). Striated 2D lattice with sub-nm 1D etch channels by controlled thermally induced phase transformations of PdSe_2_. Adv. Mater..

[50] Shautsova V, Sinha S, Hou L, Zhang Q, Tweedie M (2019). Direct laser patterning and phase transformation of 2D PdSe_2_ films for on-demand device fabrication. ACS Nano.

[51] Takabatake T, Ishikawa M, Jorda JL (1987). Superconductivity and phase relations in the Pd-Se system. J. Less Common Met..

[52] Oyedele AD, Yang S, Feng T, Haglund AV, Gu Y (2019). Defect-mediated phase transformation in anisotropic two-dimensional PdSe_2_ crystals for seamless electrical contacts. J. Am. Chem. Soc..

[53] Wang D, Luo F, Lu M, Xie X, Huang L (2019). Chemical vapor transport reactions for synthesizing layered materials and their 2D counterparts. Small.

[54] Long M, Wang Y, Wang P, Zhou X, Xia H (2019). Palladium diselenide long-wavelength infrared photodetector with high sensitivity and stability. ACS Nano.

[55] Velicky M, Donnelly GE, Hendren WR, McFarland S, Scullion D (2018). Mechanism of gold-assisted exfoliation of centimeter-sized transition-metal dichalcogenide monolayers. ACS Nano.

[56] Heyl M, Burmeister D, Schultz T, Pallasch S, Ligorio G (2020). Thermally activated gold-mediated transition metal dichalcogenide exfoliation and a unique gold-mediated transfer. Phys. Status Solidi (RRL).

[57] Desai SB, Madhvapathy SR, Amani M, Kiriya D, Hettick M (2016). Gold-mediated exfoliation of ultralarge optoelectronically-perfect monolayers. Adv. Mater..

[58] Huang Y, Pan YH, Yang R, Bao LH, Meng L (2020). Universal mechanical exfoliation of large-area 2D crystals. Nat. Commun..

[59] Zhao D, Xie S, Wang Y, Zhu H, Chen L (2019). Synthesis of large-scale few-layer PtS_2_ films by chemical vapor deposition. AIP Adv..

[60] Jia L, Wu J, Yang T, Jia B, Moss DJ (2020). Large third-order optical kerr nonlinearity in nanometer-thick PdSe_2_ 2D dichalcogenide films: implications for nonlinear photonic devices. ACS Appl. Nano Mater..

[61] Zhou J, Lin J, Huang X, Zhou Y, Chen Y (2018). A library of atomically thin metal chalcogenides. Nature.

[62] Zeng LH, Wu D, Lin SH, Xie C, Yuan HY (2019). Controlled synthesis of 2D palladium diselenide for sensitive photodetector applications. Adv. Funct. Mater..

[63] Lu LS, Chen GH, Cheng HY, Chuu CP, Lu KC (2020). Layer-dependent and in-plane anisotropic properties of low-temperature synthesized few-layer PdSe_2_ single crystals. ACS Nano.

[64] Nguyen GD, Oyedele AD, Haglund A, Ko W, Liang L (2020). Atomically precise PdSe_2_ pentagonal nanoribbons. ACS Nano.

[65] Zeng LH, Lin SH, Li ZJ, Zhang ZX, Zhang TF (2018). Fast, self-driven, air-stable, and broadband photodetector based on vertically aligned PtSe_2_/GaAs heterojunction. Adv. Funct. Mater..

[66] Hoffman AN, Gu Y, Tokash J, Woodward J, Xiao K (2020). Layer-by-layer thinning of pdse2 flakes via plasma induced oxidation and sublimation. ACS Appl. Mater. Interfaces.

[67] Q. Liang, Q. Zhang, J. Gou, T. Song, Arramel et al., Performance improvement by ozone treatment of 2D PdSe_2_. ACS Nano **14**(5), 5668–5677 (2020). 10.1021/acsnano.0c00180 10.1021/acsnano.0c00180 32364379

[68] Di Bartolomeo A, Urban F, Pelella A, Grillo A, Passacantando M (2020). Electron irradiation of multilayer PdSe_2_ field effect transistors. Nanotechnology.

[69] Hassan A, Guo Y, Wang Q (2020). Performance of the pentagonal PdSe_2_ sheet as a channel material in contact with metal surfaces and graphene. ACS Appl. Electron. Mater..

[70] Di Bartolomeo A, Pelella A, Liu X, Miao F, Passacantando M (2019). Pressure-tunable ambipolar conduction and hysteresis in thin palladium diselenide field effect transistors. Adv. Funct. Mater..

[71] Gao J, Gao Y, Han Y, Pang J, Wang C (2020). Ultrasensitive label-free MiRNA sensing based on a flexible graphene field-effect transistor without functionalization. ACS Appl. Electron. Mater..

[72] Tankut A, Karaman M, Yildiz I, Canli S, Turan R (2015). Effect of Al vacuum annealing prior to a-Si deposition on aluminum-induced crystallization. Phys. Status Solidi A Appl. Mater. Sci..

[73] Takenobu T, Kanbara T, Akima N, Takahashi T, Shiraishi M (2005). Control of carrier density by a solution method in carbon-nanotube devices. Adv. Mater..

[74] Giubileo F, Grillo A, Iemmo L, Luongo G, Urban F (2020). Environmental effects on transport properties of PdSe_2_ field effect transistors. Mater. Today Proc..

[75] Xia GT, Huang YN, Li FJ, Wang LC, Pang JB (2020). A thermally flexible and multi-site tactile sensor for remote 3D dynamic sensing imaging. Front. Chem. Sci. Eng..

[76] Chen D, Liu Z, Li Y, Sun D, Liu X (2020). Unsymmetrical alveolate PMMA/MWCNT film as a piezoresistive E-skin with four-dimensional resolution and application for detecting motion direction and airflow rate. ACS Appl. Mater. Interfaces.

[77] Zhou Y, Wang Y, Wang K, Kang L, Peng F (2020). Hybrid genetic algorithm method for efficient and robust evaluation of remaining useful life of supercapacitors. Appl. Energy.

[78] Shang X, Li S, Wang K, Teng X, Wang X (2019). MnSe_2_/Se composite nanobelts as an improved performance anode for lithium storage. Int. J. Electrochem. Sci..

[79] Bu C, Li F, Yin K, Pang J, Wang L (2020). Research progress and prospect of triboelectric nanogenerators as self-powered human body sensors. ACS Appl. Electron. Mater..

[80] Dhanabalan SC, Ponraj JS, Zhang H, Bao Q (2016). Present perspectives of broadband photodetectors based on nanobelts, nanoribbons, nanosheets and the emerging 2D materials. Nanoscale.

[81] Di Bartolomeo A, Pelella A, Urban F, Grillo A, Iemmo L (2020). Field emission in ultrathin PdSe_2_ back-gated transistors. Adv. Electron. Mater..

[82] Zhuo R, Zeng L, Yuan H, Wu D, Wang Y (2018). In-situ fabrication of PtSe_2_/GaN heterojunction for self-powered deep ultraviolet photodetector with ultrahigh current on/off ratio and detectivity. Nano Res..

[83] Buscema M, Groenendijk DJ, Blanter SI, Steele GA, van der Zant HS (2014). Fast and broadband photoresponse of few-layer black phosphorus field-effect transistors. Nano Lett..

[84] Wan X, Xu Y, Guo H, Shehzad K, Ali A (2017). A self-powered high-performance graphene/silicon ultraviolet photodetector with ultra-shallow junction: breaking the limit of silicon?. NPJ 2D Mater. Appl..

[85] Zhuo R, Wang Y, Wu D, Lou Z, Shi Z (2018). High-performance self-powered deep ultraviolet photodetector based on MoS_2_/GaN p–n heterojunction. J. Mater. Chem. C.

[86] Mukhokosi EP, Krupanidhi SB, Nanda KK (2017). Band gap engineering of hexagonal SnSe_2_ nanostructured thin films for infra-red photodetection. Sci. Rep..

[87] Luo LB, Wang D, Xie C, Hu JG, Zhao XY (2019). PdSe_2_ multilayer on germanium nanocones array with light trapping effect for sensitive infrared photodetector and image sensing application. Adv. Funct. Mater..

[88] Yang Y, Liu SC, Wang X, Li Z, Zhang Y (2019). Polarization-sensitive ultraviolet photodetection of anisotropic 2D GeS_2_. Adv. Funct. Mater..

[89] Chu F, Chen M, Wang Y, Xie Y, Liu B (2018). A highly polarization sensitive antimonene photodetector with a broadband photoresponse and strong anisotropy. J. Mater. Chem. C.

[90] Venuthurumilli PK, Ye PD, Xu X (2018). Plasmonic Resonance enhanced polarization-sensitive photodetection by black phosphorus in near infrared. ACS Nano.

[91] Yang Y, Liu SC, Yang W, Li Z, Wang Y (2018). Air-stable in-plane anisotropic GeSe_2_ for highly polarization-sensitive photodetection in short wave region. J. Am. Chem. Soc..

[92] Bullock J, Amani M, Cho J, Chen Y-Z, Ahn GH (2018). Polarization-resolved black phosphorus/molybdenum disulfide mid-wave infrared photodiodes with high detectivity at room temperature. Nat. Photon..

[93] Du J, Zhang M, Guo Z, Chen J, Zhu X (2017). Phosphorene quantum dot saturable absorbers for ultrafast fiber lasers. Sci. Rep..

[94] Ma YF, Zhang SC, Din SJ, Liu XX, Yu X (2020). Passively Q-switched Nd:GdLaNbO_4_ laser based on 2D PdSe_2_ nanosheet. Opt. Laser Technol..

[95] Ma YF, Peng ZF, Ding SJ, Sun HY, Peng F (2019). Two-dimensional WS_2_ nanosheet based passively Q-switched Nd:GdLaNbO_4_ laser. Opt. Laser Technnol..

[96] Cheng PK, Tang CY, Ahmed S, Qiao J, Zeng LH (2021). Utilization of group 10 2D TMDs-PdSe2 as a nonlinear optical material for obtaining switchable laser pulse generation modes. Nanotechnology.

[97] Pang J, Bachmatiuk A, Yin Y, Trzebicka B, Zhao L (2018). Applications of phosphorene and black phosphorus in energy conversion and storage devices. Adv. Energy Mater..

[98] Pang J, Mendes RG, Bachmatiuk A, Zhao L, Ta HQ (2019). Applications of 2D MXenes in energy conversion and storage systems. Chem. Soc. Rev..

[99] Olszowska K, Pang J, Wrobel PS, Zhao L, Ta HQ (2017). Three-dimensional nanostructured graphene: synthesis and energy, environmental and biomedical applications. Synth. Met..

[100] Zhou J, Chen H, Zhang X, Chi K, Cai Y (2021). Substrate dependence on (Sb_4_Se_6_)n ribbon orientations of antimony selenide thin films: morphology, carrier transport and photovoltaic performance. J. Alloys Compd..

[101] Shu F, Wang M, Pang J, Yu P (2019). A free-standing superhydrophobic film for highly efficient removal of water from turbine oil. Front. Chem. Sci. Eng..

[102] Wang K, Pang J, Li L, Zhou S, Li Y (2018). Synthesis of hydrophobic carbon nanotubes/reduced graphene oxide composite films by flash light irradiation. Front. Chem. Sci. Eng..

[103] Yin Y, Pang J, Wang J, Lu X, Hao Q (2019). Graphene-activated optoplasmonic nanomembrane cavities for photodegradation detection. ACS Appl. Mater. Interfaces.

[104] Liang F-X, Wang J-Z, Zhang Z-X, Wang Y-Y, Gao Y (2017). Broadband, ultrafast, self-driven photodetector based on Cs-doped FAPbI_3_ perovskite thin film. Adv. Opt. Mater..

[105] Long M, Gao A, Wang P, Xia H, Ott C (2017). Room temperature high-detectivity mid-infrared photodetectors based on black arsenic phosphorus. Sci. Adv..

[106] Yu X, Yu P, Wu D, Singh B, Zeng Q (2018). Atomically thin noble metal dichalcogenide: a broadband mid-infrared semiconductor. Nat. Commun..

[107] Hsu AL, Herring PK, Gabor NM, Ha S, Shin YC (2015). Graphene-based thermopile for thermal imaging applications. Nano Lett..

[108] Piotrowski J, Rogalski A (2004). Uncooled long wavelength infrared photon detectors. Infrared Phys. Technol..

[109] Cao Y, Zhu X, Chen H, Zhang X, Zhouc J (2019). Towards high efficiency inverted Sb_2_Se_3_ thin film solar cells. Sol. Energy Mater. Sol. Cells.

[110] Cao Y, Zhu X, Jiang J, Liu C, Zhou J (2020). Rotational design of charge carrier transport layers for optimal antimony trisulfide solar cells and its integration in tandem devices. Sol. Energy Mater. Sol. Cells.

[111] Jiang J, Meng F, Cheng Q, Wang A, Chen Y (2020). Low lattice mismatch InSe–Se vertical van der Waals heterostructure for high-performance transistors via strong fermi-level depinning. Small Methods.

[112] Jiang J, Meng F, Cheng Q, Wang A, Chen Y (2020). Low lattice mismatch InSe–Se vertical van der Waals heterostructure for high-performance transistors via strong fermi-level depinning (Small Methods 8/2020). Small Methods.

[113] Wu C-C, Jariwala D, Sangwan VK, Marks TJ, Hersam MC (2013). Elucidating the photoresponse of ultrathin MoS_2_ field-effect transistors by scanning photocurrent microscopy. J. Phys. Chem. Lett..

[114] Xue F, Chen L, Chen J, Liu J, Wang L (2016). p-Type MoS_2_ and n-type ZnO diode and its performance enhancement by the piezophototronic effect. Adv. Mater..

[115] Li D, Chen M, Sun Z, Yu P, Liu Z (2017). Two-dimensional non-volatile programmable p–n junctions. Nat. Nanotechnol..

[116] Zhang X, Grajal J, Vazquez-Roy JL, Radhakrishna U, Wang X (2019). Two-dimensional MoS_2_-enabled flexible rectenna for Wi-Fi-band wireless energy harvesting. Nature.

[117] Afzal AM, Dastgeer G, Iqbal MZ, Gautam P, Faisal MM (2020). High-performance p-BP/n-PdSe_2_ near-infrared photodiodes with a fast and gate-tunable photoresponse. ACS Appl. Mater. Interfaces.

[118] Leñero-Bardallo JA, Carmona-Galán R, Rodríguez-Vázquez A (2018). Applications of event-based image sensors—review and analysis. Int. J. Circ. Theor. Appl..

[119] Liang FX, Zhao XY, Jiang JJ, Hu JG, Xie WQ (2019). Light confinement effect induced highly sensitive, self-driven near-infrared photodetector and image sensor based on multilayer PdSe_2_ /pyramid Si heterojunction. Small.

[120] Ibrahim I, Kalbacova J, Engemaier V, Pang JB, Rodriguez RD (2015). Confirming the dual role of etchants during the enrichment of semiconducting single wall carbon nanotubes by chemical vapor deposition. Chem. Mater..

[121] Pang J, Mendes RG, Wrobel PS, Wlodarski MD, Ta HQ (2017). Self-terminating confinement approach for large-area uniform monolayer graphene directly over Si/SiOx by chemical vapor deposition. ACS Nano.

[122] Pang J, Bachmatiuk A, Ibrahim I, Fu L, Placha D (2015). CVD growth of 1D and 2D sp^2^ carbon nanomaterials. J. Mater. Sci..

[123] Soni A, Zhao L, Ta HQ, Shi Q, Pang J (2018). Facile graphitization of silicon nano-particles with ethanol based chemical vapor deposition. Nano-Struct. Nano-Objects.

[124] Sun B, Pang J, Cheng Q, Zhang S, Zhang C (2021). Synthesis of wafer-scale graphene with chemical vapor deposition for electronic device applications. Adv. Mater. Technol..

[125] Martynkova GS, Becerik F, Placha D, Pang J, Akbulut H (2019). Effect of milling and annealing on carbon-silver system. J. Nanosci. Nanotechnol..

[126] Rummeli MH, Gorantla S, Bachmatiuk A, Phieler J, Geissler N (2013). On the role of vapor trapping for chemical vapor deposition (CVD) grown graphene over copper. Chem. Mater..

[127] Pang JB, Bachmatiuk A, Fu L, Yan CL, Zeng MQ (2015). Oxidation as a means to remove surface contaminants on Cu foil prior to graphene growth by chemical vapor deposition. J. Phys. Chem. C.

[128] Pang JB, Bachmatiuk A, Fu L, Mendes RG, Libera M (2015). Direct synthesis of graphene from adsorbed organic solvent molecules over copper. RSC Adv..

[129] Santhosh NM, Filipič G, Kovacevic E, Jagodar A, Berndt J (2020). N-graphene nanowalls via plasma nitrogen incorporation and substitution: the experimental evidence. Nano-Micro Lett..

[130] Mendes RG, Pang J, Bachmatiuk A, Ta HQ, Zhao L (2019). Electron-driven in situ transmission electron microscopy of 2D transition metal dichalcogenides and their 2D heterostructures. ACS Nano.

[131] Zhang D, Liu T, Cheng J, Cao Q, Zheng G (2019). Lightweight and high-performance microwave absorber based on 2D WS_2_–RGO heterostructures. Nano-Micro Lett..

[132] K. Persson, Materials Data on PdSe_2_ (SG:61) by Materials Project. 10.17188/1199960

[133] Feng L-Y, Villaos RAB, Huang Z-Q, Hsu C-H, Chuang F-C (2020). Layer-dependent band engineering of Pd dichalcogenides: a first-principles study. New J. Phys..

[134] K. Persson, Materials Data on PdS_2_ (SG:61) by Materials Project. 10.17188/1189716

[135] K. Persson. Materials Data on Te_2_Pd (SG:164) by Materials Project. 10.17188/1307608

[136] Anemone G, Casado Aguilar P, Garnica M, Calleja F, Al Taleb A (2021). Electron–phonon coupling in superconducting 1T-PdTe_2_. NPJ 2D Mater. Appl..

[137] Madhu RN (2011). Singh, Palladium selenides as active methanol tolerant cathode materials for direct methanol fuel cell. Int. J. Hydrogen Energy.

[138] Qin D, Yan P, Ding G, Ge X, Song H (2018). Monolayer PdSe_2_: a promising two-dimensional thermoelectric material. Sci. Rep..

[139] Zhang G, Amani M, Chaturvedi A, Tan C, Bullock J (2019). Optical and electrical properties of two-dimensional palladium diselenide. Appl. Phys. Lett..

[140] Hoffman AN, Gu Y, Liang L, Fowlkes JD, Xiao K (2019). Exploring the air stability of PdSe_2_ via electrical transport measurements and defect calculations. NPJ 2D Mater. Appl..

[141] Fang H, Hu W (2017). Photogating in low dimensional photodetectors. Adv. Sci..

[142] Miro P, Ghorbani-Asl M, Heine T (2014). Two dimensional materials beyond MoS_2_: noble-transition-metal dichalcogenides. Angew. Chem. Int. Ed..

[143] Li L, Wang W, Chai Y, Li H, Tian M (2017). Few-layered PtS_2_ phototransistor on h-BN with high gain. Adv. Funct. Mater..

[144] Xu H, Huang HP, Fei H, Feng J, Fuh HR (2019). Strategy for fabricating wafer-scale platinum disulfide. ACS Appl. Mater. Interfaces.

[145] Zhang E, Jin Y, Yuan X, Wang W, Zhang C (2015). ReS_2_-based field-effect transistors and photodetectors. Adv. Funct. Mater..

[146] Shim J, Oh A, Kang DH, Oh S, Jang SK (2016). High-performance 2D rhenium disulfide (ReS2) transistors and photodetectors by oxygen plasma treatment. Adv. Mater..

[147] Zhang E, Wang P, Li Z, Wang H, Song C (2016). Tunable ambipolar polarization-sensitive photodetectors based on high-anisotropy ReSe_2_ nanosheets. ACS Nano.

[148] Hafeez M, Gan L, Li H, Ma Y, Zhai T (2016). Chemical vapor deposition synthesis of ultrathin hexagonal ReSe_2_ flakes for anisotropic raman property and optoelectronic application. Adv. Mater..

[149] Feng W, Wu J-B, Li X, Zheng W, Zhou X (2015). Ultrahigh photo-responsivity and detectivity in multilayer InSe nanosheets phototransistors with broadband response. J. Mater. Chem. C.

[150] Dai M, Chen H, Feng R, Feng W, Hu Y (2018). A dual-band multilayer InSe self-powered photodetector with high performance induced by surface plasmon resonance and asymmetric Schottky junction. ACS Nano.

[151] Ye J, Soeda S, Nakamura Y, Nittono O (1998). Crystal structures and phase transformation in In_2_Se_3_ compound semiconductor. Jpn. J. Appl. Phys..

[152] Feng W, Gao F, Hu Y, Dai M, Liu H (2018). Phase-engineering-driven enhanced electronic and optoelectronic performance of multilayer In2Se3 nanosheets. ACS Appl. Mater. Interfaces.

[153] Jacobs-Gedrim RB, Shanmugam M, Jain N, Durcan CA, Murphy MT (2014). Extraordinary photoresponse in two-dimensional In(2)Se(3) nanosheets. ACS Nano.

[154] Amani M, Regan E, Bullock J, Ahn GH, Javey A (2017). Mid-wave infrared photoconductors based on black phosphorus-arsenic alloys. ACS Nano.

[155] Zheng D, Fang H, Long M, Wu F, Wang P (2018). High-performance near-infrared photodetectors based on p-type SnX (X = S, Se) nanowires grown via chemical vapor deposition. ACS Nano.

[156] Su G, Hadjiev VG, Loya PE, Zhang J, Lei S (2015). Chemical vapor deposition of thin crystals of layered semiconductor SnS_2_ for fast photodetection application. Nano Lett..

[157] Xia F, Mueller T, Lin YM, Valdes-Garcia A, Avouris P (2009). Ultrafast graphene photodetector. Nat. Nanotechnol..

[158] Kim BJ, Jang H, Lee SK, Hong BH, Ahn JH (2010). High-performance flexible graphene field effect transistors with ion gel gate dielectrics. Nano Lett..

[159] Polat EO, Mercier G, Nikitskiy I, Puma E, Galan T (2019). Flexible graphene photodetectors for wearable fitness monitoring. Sci. Adv..

[160] Yu X, Dong Z, Liu Y, Liu T, Tao J (2016). A high performance, visible to mid-infrared photodetector based on graphene nanoribbons passivated with HfO_2_. Nanoscale.

[161] Zeng L, Tao L, Tang C, Zhou B, Long H (2016). High-responsivity UV-Vis photodetector based on transferable WS_2_ film deposited by magnetron sputtering. Sci. Rep..

[162] Jiang J, Zhang Q, Wang A, Zhang Y, Meng F, Zhang C, Feng X, Feng Y, Gu L, Liu H, Han L (2019). A facile and effective method for patching sulfur vacancies of WS2 via nitrogen plasma treatment. Small.

[163] Wang Q, Zhang Q, Zhao X, Zheng YJ, Wang J (2019). High-energy gain upconversion in monolayer tungsten disulfide photodetectors. Nano Lett..

[164] Zhang W, Chiu MH, Chen CH, Chen W, Li LJ (2014). Role of metal contacts in high-performance phototransistors based on WSe_2_ monolayers. ACS Nano.

[165] Zhou H, Wang C, Shaw JC, Cheng R, Chen Y (2015). Large area growth and electrical properties of p-type WSe_2_ atomic layers. Nano Lett..

[166] Chen J, Wang Q, Sheng Y, Cao G, Yang P (2019). High-performance WSe_2_ photodetector based on a laser-induced p–n junction. ACS Appl. Mater. Interfaces.

[167] Lee HS, Min SW, Chang YG, Park MK, Nam T (2012). MoS(2) nanosheet phototransistors with thickness-modulated optical energy gap. Nano Lett..

[168] Zhou YH, An HN, Gao C, Zheng ZQ, Wang B (2019). UV–Vis-NIR photodetector based on monolayer MoS_2_. Mater. Lett..

[169] Wang W, Klots A, Prasai D, Yang Y, Bolotin KI (2015). Hot electron-based near-infrared photodetection using bilayer MoS_2_. Nano Lett..

[170] Jung C, Kim SM, Moon H, Han G, Kwon J (2015). Highly crystalline CVD-grown multilayer MoSe_2_ thin film transistor for fast photodetector. Sci. Rep..

[171] Coehoorn R, Haas C, de Groot RA (1987). Electronic structure of MoSe_2_, MoS_2_, and WSe_2_. II. The nature of the optical band gaps. Phys. Rev. B.

[172] Ko PJ, Abderrahmane A, Kim NH, Sandhu A (2017). High-performance near-infrared photodetector based on nano-layered MoSe_2_. Semicond. Sci. Technol..

[173] Tran V, Soklaski R, Liang Y, Yang L (2014). Layer-controlled band gap and anisotropic excitons in few-layer black phosphorus. Phys. Rev. B.

[174] Guo Q, Pospischil A, Bhuiyan M, Jiang H, Tian H (2016). Black phosphorus mid-infrared photodetectors with high gain. Nano Lett..

[175] Qiao J, Kong X, Hu ZX, Yang F, Ji W (2014). High-mobility transport anisotropy and linear dichroism in few-layer black phosphorus. Nat. Commun..

[176] Wang J, Rousseau A, Eizner E, Phaneuf-L’Heureux A-L, Schue L (2019). Spectral responsivity and photoconductive gain in thin film black phosphorus photodetectors. ACS Photon..

[177] Zhou X, Hu X, Jin B, Yu J, Liu K (2018). Highly anisotropic GeSe nanosheets for phototransistors with ultrahigh photoresponsivity. Adv. Sci..

[178] Jia C, Wu D, Wu EP, Guo JW, Zhao ZH (2019). A self-powered high-performance photodetector based on a MoS_2_/GaAs heterojunction with high polarization sensitivity. J. Mater. Chem. C..

[179] Chai R, Chen Y, Zhong M, Yang H, Yan F (2020). Non-layered ZnSb nanoplates for room temperature infrared polarized photodetectors. J. Mater. Chem. C.

[180] Deng S, Tao ML, Mei J, Li M, Zhang Y (2019). Optical and piezoelectric properties of strained orthorhombic PdS_2_. IEEE Trans. Nanotechnol..

[181] Deng Y, Luo Z, Conrad NJ, Liu H, Gong Y (2014). Black phosphorus-monolayer MoS_2_ van der Waals heterojunction p–n diode. ACS Nano.

[182] Yan F, Zhao L, Patane A, Hu P, Wei X (2017). Fast, multicolor photodetection with graphene-contacted p-GaSe/n-InSe van der Waals heterostructures. Nanotechnology.

[183] Chen X, Chen H, Wang Z, Shan Y, Zhang DW (2018). Analysis of the relationship between the contact barrier and rectification ratio in a two-dimensional P–N heterojunction. Semicond. Sci. Technol..

[184] Murali K, Dandu M, Das S, Majumdar K (2018). Gate-tunable WSe_2_/SnSe_2_ backward diode with ultrahigh-reverse rectification ratio. ACS Appl. Mater. Interfaces.

[185] Khan MA, Rathi S, Lim D, Yun SJ, Youn D-H (2018). Gate tunable self-biased diode based on few layered MoS_2_ and WSe_2_. Chem. Mater..

[186] Yang Z, Liao L, Gong F, Wang F, Wang Z (2018). WSe_2_/GeSe heterojunction photodiode with giant gate tunability. Nano Energy.

[187] Lan C, Li C, Wang S, He T, Jiao T (2016). Zener tunneling and photoresponse of a WS_2_/Si van der Waals heterojunction. ACS Appl. Mater. Interfaces.

[188] Chu J, Wang F, Yin L, Lei L, Yan C (2017). High-performance ultraviolet photodetector based on a few-layered 2D NiPS_3_ nanosheet. Adv. Funct. Mater..

[189] Ye L, Li H, Chen Z, Xu J (2016). Near-infrared photodetector based on MoS_2_/black phosphorus heterojunction. ACS Photon..

[190] Zhang Y, Yu Y, Mi L, Wang H, Zhu Z (2016). In situ fabrication of vertical multilayered MoS_2_/Si homotype heterojunction for high-speed visible-near-infrared photodetectors. Small.

[191] Liu Q, Cook B, Gong M, Gong Y, Ewing D (2017). Printable transfer-free and wafer-size MoS_2_/graphene van der Waals heterostructures for high-performance photodetection. ACS Appl. Mater. Interfaces.

[192] Gundimeda A, Krishna S, Aggarwal N, Sharma A, Sharma ND (2017). Fabrication of non-polar GaN based highly responsive and fast UV photodetector. Appl. Phys. Lett..

[193] Wang P, Liu S, Luo W, Fang H, Gong F (2017). Arrayed van der Waals broadband detectors for dual-band detection. Adv. Mater..

[194] Um DS, Lee Y, Lim S, Park J, Yen WC (2016). InGaAs nanomembrane/si van der waals heterojunction photodiodes with broadband and high photoresponsivity. ACS Appl. Mater. Interfaces.

[195] Zheng W, Lin R, Zhu Y, Zhang Z, Ji X (2018). Vacuum ultraviolet photodetection in two-dimensional oxides. ACS Appl. Mater. Interfaces.

[196] Zeng LH, Wang MZ, Hu H, Nie B, Yu YQ (2013). Monolayer graphene/germanium Schottky junction as high-performance self-driven infrared light photodetector. ACS Appl. Mater. Interfaces.

[197] Li X, Zhu M, Du M, Lv Z, Zhang L (2016). High detectivity graphene-silicon heterojunction photodetector. Small.

[198] Zhang K, Fang X, Wang Y, Wan Y, Song Q (2017). Ultrasensitive near-infrared photodetectors based on a graphene-MoTe_2_-graphene vertical van der Waals heterostructure. ACS Appl. Mater. Interfaces.

[199] Lan Y-S, Chen X-R, Hu C-E, Cheng Y, Chen Q-F (2019). Penta-PdX_2_ (X = S, Se, Te) monolayers: promising anisotropic thermoelectric materials. J. Mater. Chem. A.

